# Supplementary material for: Dissipationless transport signature of topological nodal lines
Source: Nat Commun. 2025 Jul 21;16:6711. doi: 10.1038/s41467-025-61059-8 (PMC12280140; doi:10.1038/s41467-025-61059-8)
Supplement: Supplementary file 1 — Supplementary Information [file 41467_2025_61059_MOESM1_ESM.pdf]

Dissipationless transport signature of topological nodal lines

– Supplementary Information –

Arthur Veyrat,[1,2,3,*] Klaus Koepernik,[1,2] Louis Veyrat,[1,2,4] Grigory Shipunov,[1,2] Iryna Kovalchuk,[1,5] Saicharan Aswartham,[1,2] Jiang Qu,[1,2] Ankit Kumar,[1,2] Michele Ceccardi,[6,7] Federico Caglieris,[7] Nicolás Pérez Rodríguez,[1,2] Romain Giraud,[1,2,8] Bernd Büchner,[1,2,9] Jeroen van den Brink,[1,2,9] Carmine Ortix,[10,†] and Joseph Dufouleur[1,2,11,‡]

[1] *Leibniz Institute for Solid State and Materials Research (IFW Dresden), Helmholtzstraße 20, D-01069 Dresden, Germany*
[2] *Würzburg-Dresden Cluster of Excellence ct.qmat, Dresden, Germany*
[3] *Laboratoire de Physique des Solides (LPS Orsay), 510 Rue André Rivière, 91400 Orsay, France*
[4] *CNRS, Laboratoire National des Champs Magnétiques Intenses, Université Grenoble-Alpes, Université Toulouse 3, INSA-Toulouse, EMFL, 31400 Toulouse, France*
[5] *Kyiv Academic University, 03142 Kyiv, Ukraine*
[6] *Department of Physics, University of Genoa, 16146 Genoa, Italy*
[7] *CNR-SPIN Institute, 16152 Genoa, Italy*
[8] *Université Grenoble Alpes, CNRS, CEA, Grenoble-INP, Spintec, F-38000 Grenoble, France*
[9] *Department of Physics, TU Dresden, D-01062 Dresden, Germany*
[10] *Dipartimento di Fisica "E. R. Caianiello", Universitá di Salerno, IT-84084 Fisciano (SA), Italy*
[11] *Center for Transport and Devices, TU Dresden, D-01069 Dresden, Germany*

CONTENTS

A. Resistivity in the anomalous planar Hall effect 2
B. Residues extraction for APHE vs B 4
C. Residues extraction for APHE vs T 6
D. APHE vs B 8
E. APHE vs T 10
F. PHE and APHE at 126nm 12
G. PHE and APHE at 320nm 14
H. Residual Resistance Ratio 16
I. The Weyl point geometry of band 48 17
J. Chern signal 18
K. Band 47 21
L. Lattice structure 21
M. TNL conversion into Weyl Nodes: Model Hamiltonian 22
N. TNL gap in magnetic field and thermal activation 22

References 33

* arthur.veyrat@universite-paris-saclay.fr
† cortix@unisa.it
‡ j.dufouleur@ifw-dresden.de

A. Resistivity in the anomalous planar Hall effect

It is expected from the theory [1] that the conductivity generated by the anomalous planar Hall effect (APHE) follows the angular dependence

$$\begin{aligned}\sigma_{xx}^{\mathrm{APHE}}(\varphi) &= 0,\\ \sigma_{yx}^{\mathrm{APHE}}(\varphi) &= \Delta\sigma_{yx}\cos 3\varphi.\end{aligned} \tag{1}$$

In order to obtain the angular dependence of the resistivity associated with the APHE, we assume an underlying constant (Drude) longitudinal conductivity $\sigma_{xx} = \sigma_D$, so that the overall conductivity matrix becomes $\sigma = \begin{pmatrix} \sigma_D & \sigma_{xy} \\ -\sigma_{xy} & \sigma_D \end{pmatrix}$, with $\sigma_{xy} = \sigma_{xy}^{\mathrm{APHE}}(\varphi)$ from eq. S1. By inverting the conductivity matrix, we obtain the resistivity matrix $\rho = \sigma^{-1} = \begin{pmatrix} \rho_{xx} & \rho_{xy} \\ -\rho_{xy} & \rho_{xx} \end{pmatrix}$, with

$$\begin{aligned}\rho_{xx} &= \frac{\sigma_D}{\sigma_D^2 + \sigma_{xy}^2} = \frac{1}{\sigma_D(1 + (\sigma_{xy}/\sigma_D)^2)},\\ \rho_{xy} &= -\frac{\sigma_{xy}}{\sigma_D^2 + \sigma_{xy}^2} = \frac{\sigma_{xy}/\sigma_D}{\sigma_D(1 + (\sigma_{xy}/\sigma_D)^2)}.\end{aligned} \tag{2}$$

Here, we can see that the APHE has a more complicated effect on the resistivity than on the conductivity, with both ρ_{xx} and ρ_{xy} having $2\pi/3$-periodic components in φ. The shape of the oscillation depends strongly on the relative amplitude $r = \Delta\sigma_{xy}/\sigma_D$ of the AHC oscillation with respect to the longitudinal conductivity (see Fig. 1.A,B,C). For $r \ll 1$, both ρ_{xx} and ρ_{xy} tend towards sine waves of period $\pi/3$ and $2\pi/3$, respectively. The amplitudes of the oscillations of ρ_{xx} and ρ_{xy} (defined as $\Delta\rho = [\max(\rho) - \min(\rho)]/2$) depend on the ratio r as r^3 and r^2, respectively, meaning that for $r \ll 1$, the oscillations in ρ_{xx} will be much lower than those in ρ_{xy} (see Fig. 1.F).

As the ratio r is proportional to the angular mean value $\overline{\rho}_{xx}$ of ρ_{xx}, this means that for a given amplitude of the oscillations in AHC, the APHE in resistivities will deviate from sine waves when the longitudinal resistivity is too high, and the oscillations will vanish if the longitudinal resistivity is too low. For our sample, we can roughly estimate a ratio $r = \dfrac{\Delta\sigma_{xy}}{\sigma_D} \sim 2.7 \times 10^{-3}$, by taking $\sigma_D = 1/\overline{\rho}_{xx} = 1/(R_{xx}(B = 0)/N_{\square}) \sim 1/(0.8/0.3)$ and $\Delta\sigma_{xy} = 0.001$, which gives an amplitude of the oscillations of ρ_{xy} of about $7\mu\Omega$, close to what we see at 14T (see Fig. 2.d in the main text). At this value, the oscillations in ρ_{xy} are very close to a sine wave, and the oscillations in ρ_{xx} are about 3 orders of magnitude smaller. This shows that, in our case, we can simply treat the signature of the APHE in resistivity as

$$\begin{aligned}\rho_{xx}^{\mathrm{APHE}}(\varphi) &= 0,\\ \rho_{xy}^{\mathrm{APHE}}(\varphi) &= A^{\mathrm{APHE}}\cos 3\varphi.\end{aligned} \tag{3}$$

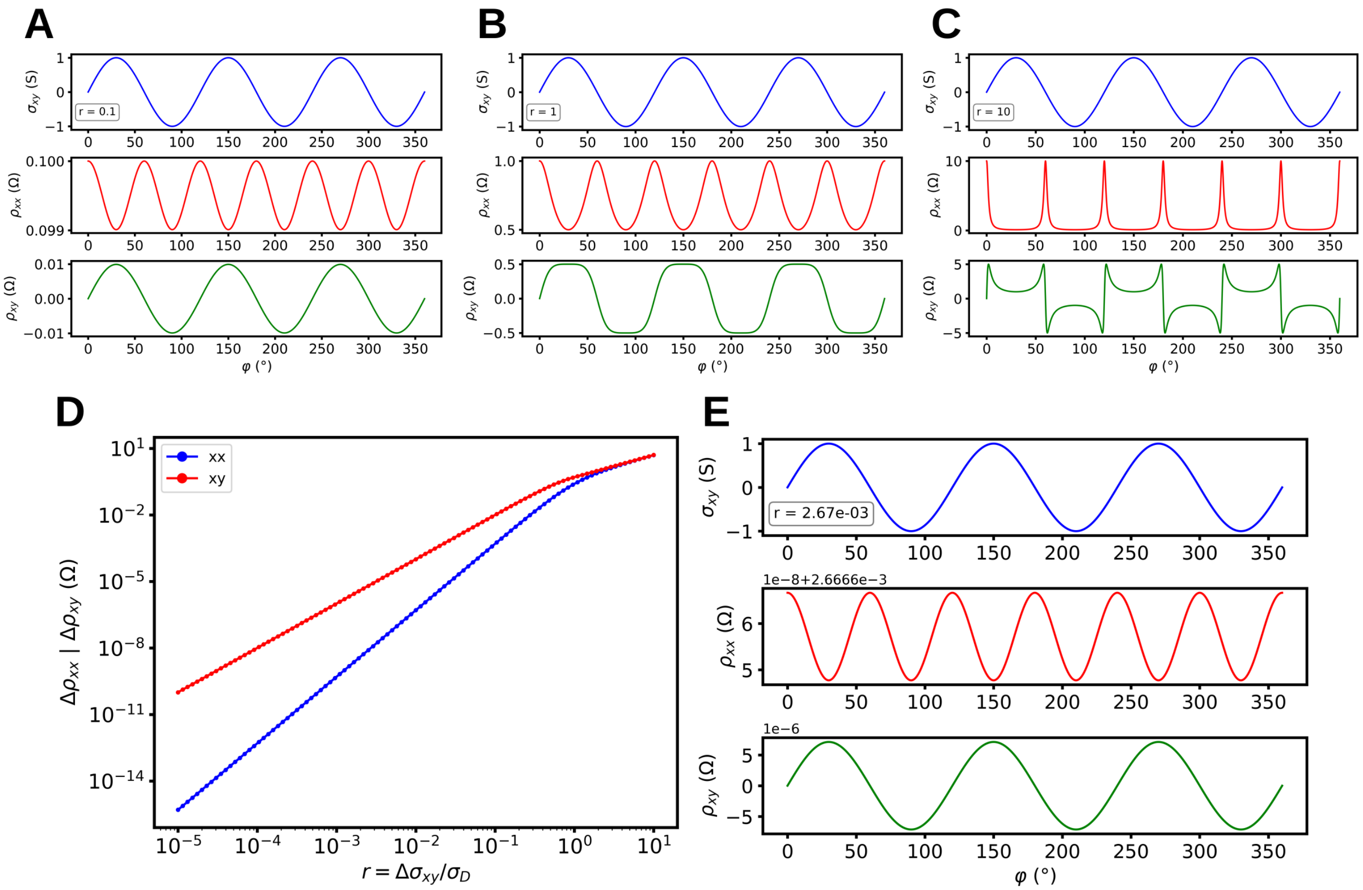

Supplementary Figure 1. Resistivitities of the APHE. a,b,c: ρ_{xx} and ρ_{xy} obtained from σ for the ratios 0.1,1 and 10. d: Amplitude of the oscillations of ρ_{xx} and ρ_{xy} with respect to the ratio r, in log scale. e: ρ_{xx} and ρ_{xy} obtained for a realistic value of $r = 2.67 \times 10^{-3}$.

B. Residues extraction for APHE vs B

In order to extract as much of the background and PHE as possible, and try to keep only the APHE, we can fit R_{xx} and R_{yx} separately at each field with a simple model:

$$\overline{R}(\varphi) = C + A^{2\pi} \cdot \cos(\varphi - \varphi_{2\pi}) + A^{\pi} \cdot \cos[2(\varphi - \varphi_{\pi})], \quad (4)$$

with C an angle-independent constant, and A^{π} and $A^{2\pi}$ the amplitudes of some π- and 2π-periodic signals, with angular origins φ_{π} and $\varphi_{2\pi}$, respectively. The π-periodic component should remove the PHE signal from the measurements, while the angle-independent and 2π-periodic components should remove the magnetoresistance signal from the perpendicular field component (respectively for the misalignment between the rotation plane and magnetic field axis, and for the misalignment of the sample's plane with the rotation plane). All the above parameters are independent between R_{xx} and R_{yx}, and are assumed to be field- or temperature-dependent. Fits of the data at each magnetic field are shown in red in Fig. 2.A-I. The residues ΔR are then obtained by subtracting the previous fit from the data: $\Delta R = R - \overline{R}$.

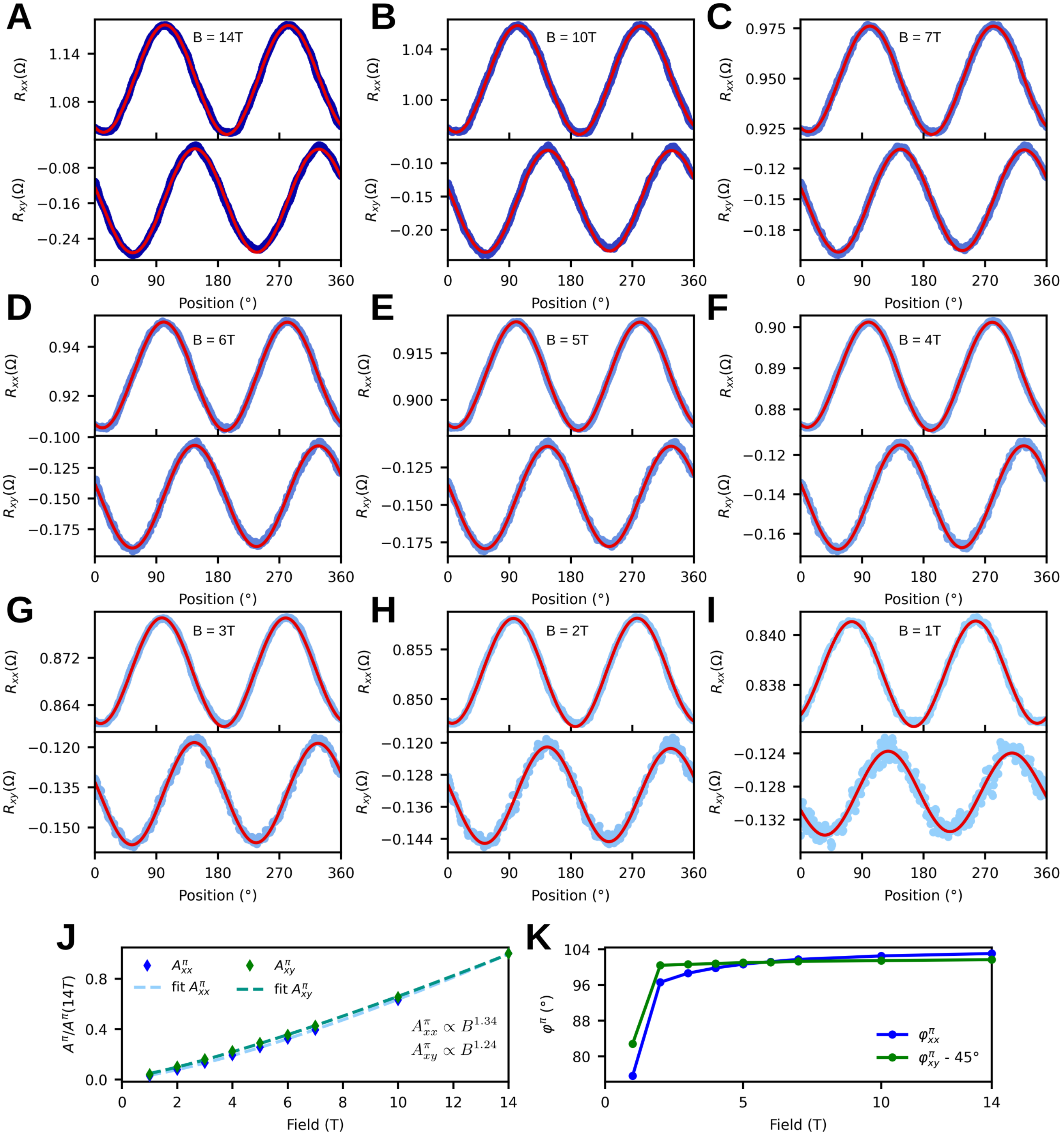

Supplementary Figure 2. A-I indicate in blue the angle dependence of R_{xx} and R_{yx} at low temperature and for a magnetic field amplitude ranging between 14T (A) and 1T (I). Fits of the data at each magnetic field are shown in red. J shows the field dependence of the amplitude of the longitudinal resistance (blue diamond) and transverse resistance (green diamond) and the fit with the associated power law (dashed lines). K indicates the value of the period obtained for the fit of the longitudinal (blue) or transverse (green) resistances.

C. Residues extraction for APHE vs T

We perform the exact same analysis as in sec. B at 14T and multiple temperatures, from 5K to 300K. The fits are shown in grey in Fig. 3.A-G.

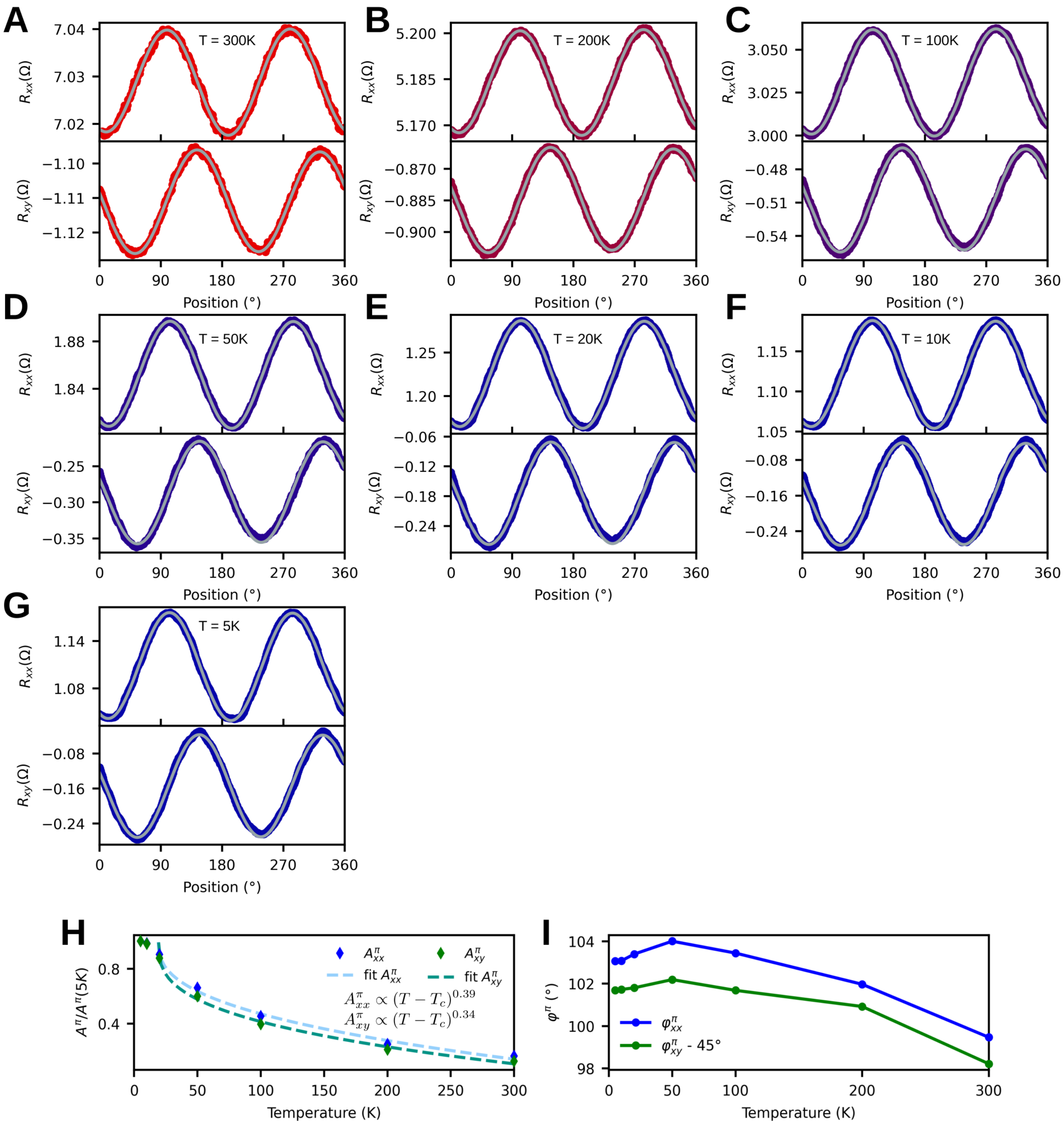

Supplementary Figure 3. A-G indicate in blue the angle dependence of R_{xx} and R_{yx} at 14T and for a temperature ranging between 300K (A) and 5K (I). Fits of the data at each magnetic field are shown in grey. H shows the temperature dependence of the amplitude of the longitudinal resistance (blue diamond) and transverse resistance (green diamond) and the fit with the associated power law (dashed lines). I indicates the value of the period obtained for the fit of the longitudinal (blue) or transverse (green) resistances.

D. APHE vs B

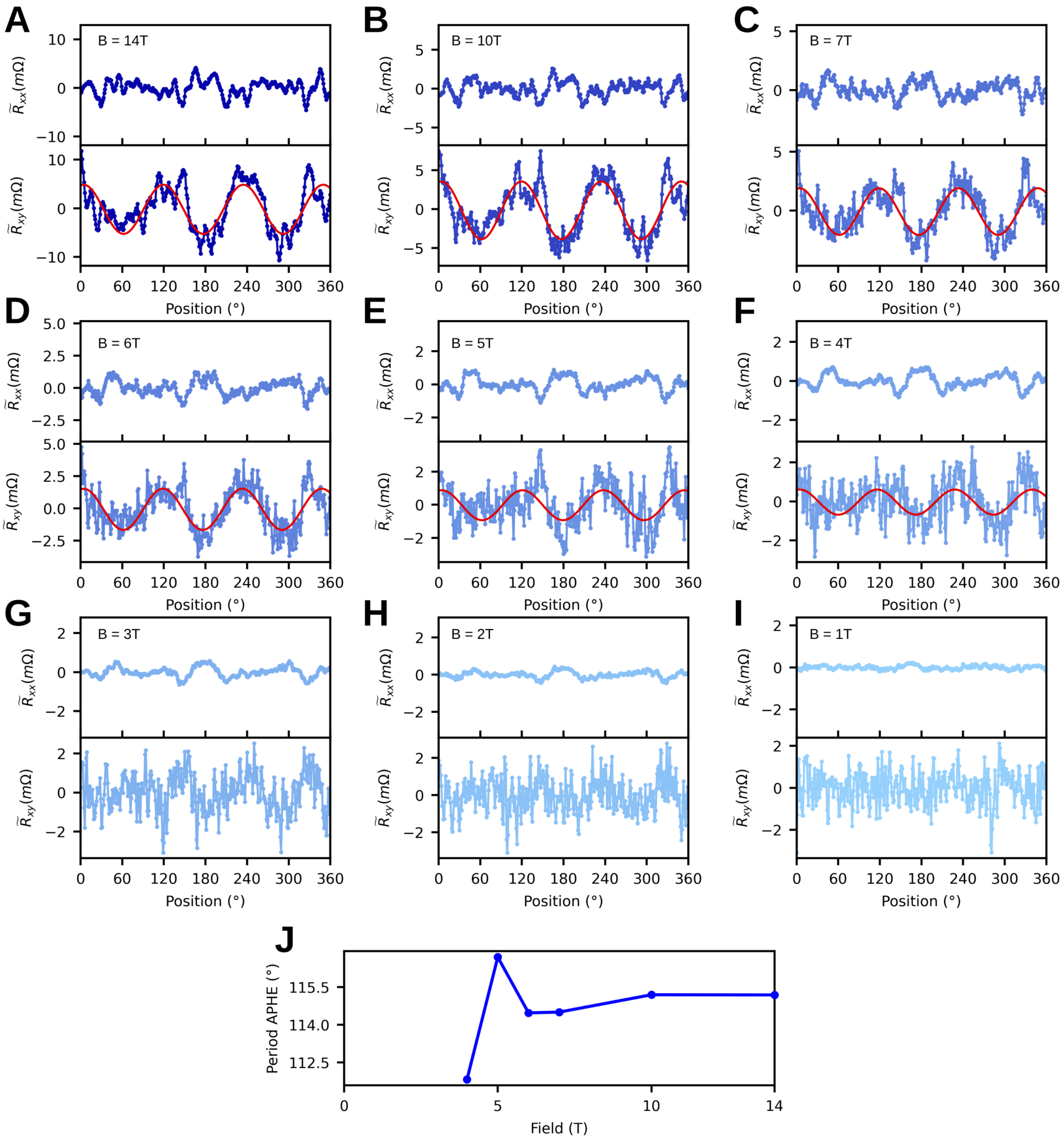

Supplementary Figure 4. A-I show in blue the longitudinal and transverse residues $\Delta R = R - \overline{R}$ obtained in sec. B at 5K for each magnetic field from 1T to 14T. The red lines correspond to a fit with Eq. 5. J indicates the magnetic field dependence of the period extracted from the fit.

Fig. 4.A-I shows the longitudinal and transverse residues $\Delta R = R - \overline{R}$ obtained in sec. B at 5K for each magnetic field from 1T to 14T. For $B \geq 4$ T, a roughly $2\pi/3$-periodic signal emerges in ΔR_{yx}, which corresponds to the APHE.

We fit this signal with the following formula:

$$\Delta R_{yx}(B,\varphi) = C(B) + A^{\mathrm{APHE}}(B)\cos[(\varphi-\varphi_0)\cdot 2\pi/P^{\mathrm{APHE}}(B)], \tag{5}$$

with C a field dependent offset ; A^{APHE} the amplitude of the APHE ; φ_0 the field-independent angular origin of the oscillation ; and P^{APHE} the angular period of the APHE, which is considered field-dependent in the analysis. The fits obtained above 4T are shown in red in Fig. 4.A-I, and fit the data very well between $120° \leq \widetilde{\varphi} \leq 360°$. The residues deviate slightly from the fit below 120°, most probably due to non-uniform movement of the mechanical rotator in this angular range at low temperature. This issue is much more visible in a second sample, D2 (126nm thick, see e.g. Fig. 6.A,B), and happens around specific positions in a reproducible manner. This low-angle deviation disappears at higher temperature (see sec. E), which also points towards a low-temperature mechanical issue of the rotator.
Fig. 4.J shows the field dependence of P^{APHE} yielded by the fit. Above 6T, it is stable at $P^{\mathrm{APHE}} \sim 115°$, or just under the 120° expected for the PHE from theory. The low-field variation of the period could be the result of lower signal/noise ratio combined with the mechanical rotation issues. The variation of A^{APHE} with field is shown in the main text in Fig. 2.g.

E. APHE vs T

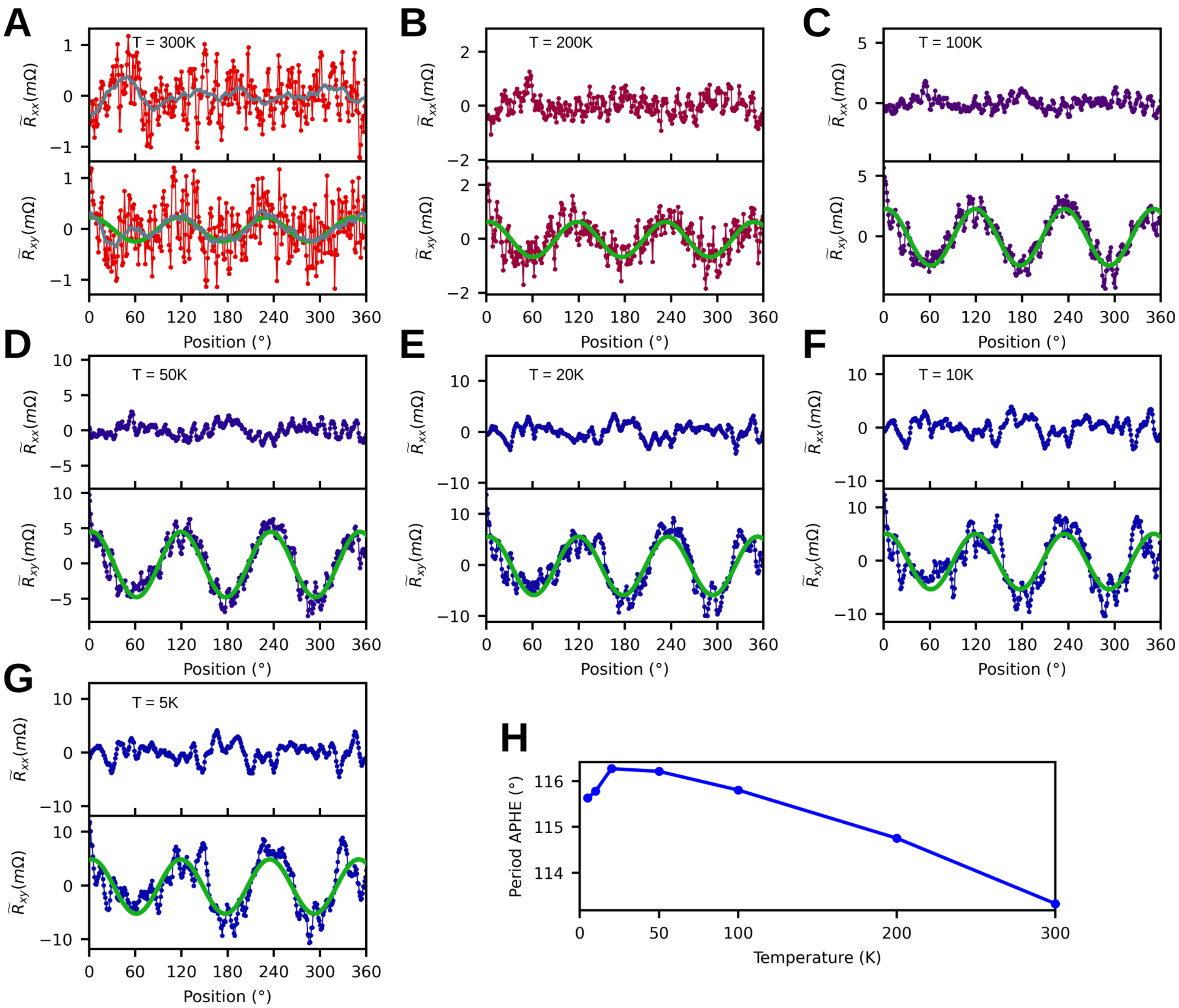

Supplementary Figure 5. A-G indicate the residues at temperature ranging between 300K and 5K at a magnetic field amplitude of 14T. The green lines correspond to a fit of the data according to the procedure described in sec. E. H indicates the E and F indicates respectively the field and temperature dependence of the PHE. G and H show respectively the field and temperature dependence of the amplitude of the longitudinal resistance (blue diamond) and transverse resistance (green diamond) and the fit with the associated power law (dashed lines).

Fig. 5.A-G shows the longitudinal and transverse residues $\Delta R = R - \overline{R}$ obtained in sec. C at 14T for each temperature from 5K to 300K (same data as in Fig. 2.d-f, main text). The raw residues are fitted with eq. 5, with the temperature as a parameter instead of the field, and the resulting fits are shown in green. The low-angle deviation from the fit observed in sec. D persist until $T \sim 50$ K, above which they are no longer visible, and the fit follows the data closely over the entire angular range.
As the temperature is increased, the amplitude of the APHE decreases (see Fig. 2.h, main text). At 300K (Fig. 5.A), the APHE is no longer visible to the naked eye because of the low signal/noise ratio. However, by applying a simple Savitzky-Golay smooth (in grey), the $2\pi/3$-periodic oscillation becomes clearly visible, and follows closely the fit of the raw data (in green). Applying the same smooth to the longitudinal residuals does not yield any similar trend.
The Savitzky-Golay smooths were done with the savgol_filter function from the scipy.signal library in Python, with 31° of window size and a polynome order of 1. The boundary conditions were set to 'wrap' (i.e. periodic) for the

transverse residues, and 'nearest' for the longitudinal residues.

F. PHE and APHE at 126nm

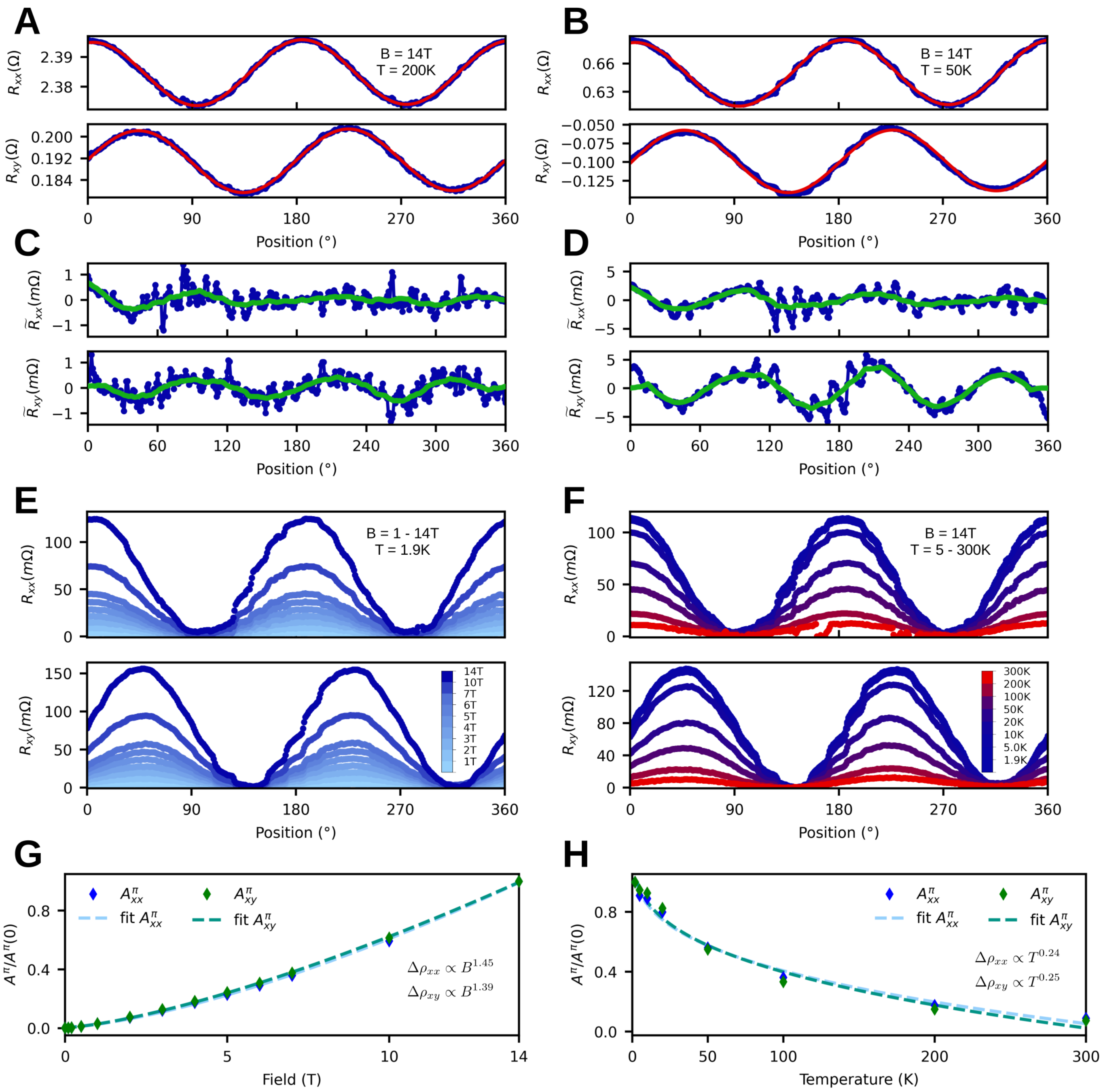

Supplementary Figure 6. A-D resistances and residues at 200K and 50K at a magnetic field amplitude of 14T. The resistance is fitted with eq. 4 (red), and the residues are smoothed with the same procedure as described in sec. E (green). E and F indicates respectively the field and temperature dependence of the PHE. G and H show respectively the field and temperature dependence of the amplitude of the longitudinal resistance (blue diamond) and transverse resistance (green diamond) and the fit with the associated power law (dashed lines).

Similar measurements and analysis as for sample $D1$ were performed on another sample, $D2$ (126nm thick). However, this sample proved more sensitive to the issues of the mechanical rotator than D1, and the APHE signal was not necessarily visible in all residues extracted, due to increased noise levels.
Fig. 6 shows the main results for sample D2. Fig. 6.A,C show the resistance and residues at 200K, 14T. The resistance is fitted with eq. 4 (red), and the residues are smoothed with the same procedure as described in sec. E

(green). Fig. 6.B,D show the same measurements at 50K, 14T. At both temperatures, the data residues show a clear oscillation corresponding to the APHE.

G. PHE and APHE at 320nm

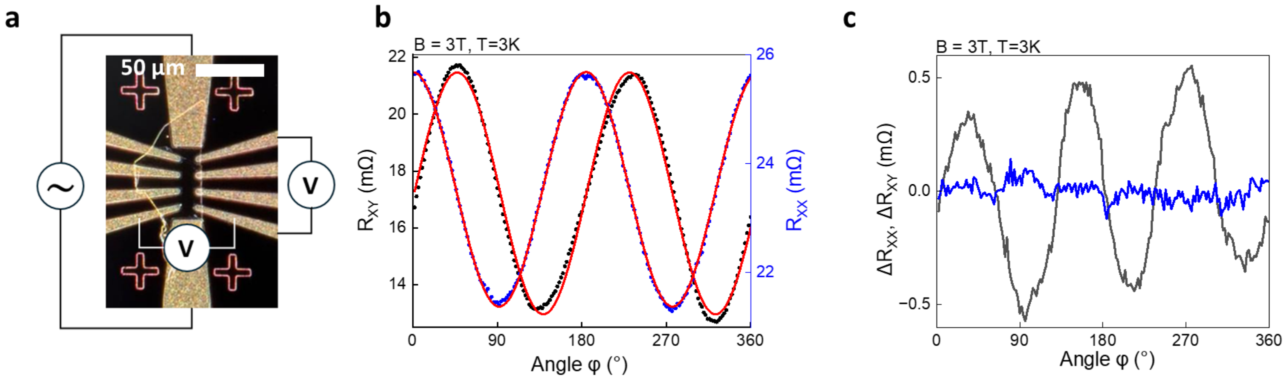

Supplementary Figure 7. **a:** Optical image of the device and schematic diagram of the measurement configurations. The Hall bar is 50μm long and 10μm large.**b**: Angular dependence of R_{yx} (black) and R_{XX} (blue) at $B = 3$ T, and $T = 3$ K. The best fits with Eq. 1(Methods) are superimposed in red. **c**: Angular dependence of the residues ΔR_{yx} (grey) and ΔR_{yx} (blue) after a background removal. Only a π-periodic signal was removed for the present analysis and a 2π-periodic signal is therefore still visible in the residues of ΔR_{yx}.

We have also exfoliated and measured a 320nm thick $PtBi_2$ flake (sample D3, see fig. 7 a). The flake was contacted by optical lithography and for such thick flake, the residual resistance ratio is much larger than for D1 or D2 : $RRR = 25$.

As already stated in the methods, the flake was measured in a different set-up and using a 3D-piezorotator. The analysis was done following equation Eq. 1in the Methods, and only a π-periodic signal was removed from the longitudinal and transverse resistances (fig. 7 b). As a result, an additional weak 2π signal can be seen in the residual of the transverse resistance. As a confirmation of the results obtained for D1 and D2, a very clear $2\pi/3$-periodic signal can be seen in the transverse resistance whereas no $2\pi/3$-periodic oscillation could be identified in the longitudinal resistance (fig. 7 c).

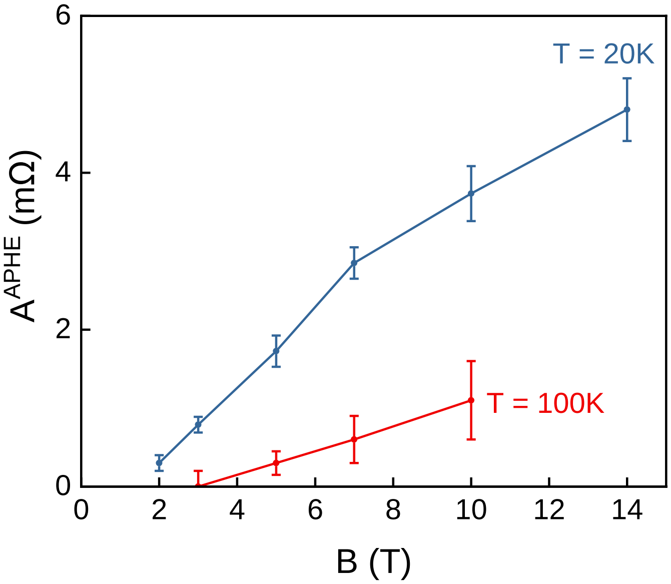

Supplementary Figure 8. Amplitude of the APHE signal as a function of the magnetic field and for $T = 20$ K (blue) and $T = 100$ K. The error bar indicates the amplitude of the noise for each measurement.

For D3, the evolution of the APHE signal was measured as a function of the magnetic field for two temperatures : $T = 20$ K and $T = 100$ K (fig. 8). A clear temperature dependence of the field onset could be evidenced, with a

higher onset seen at higher temperature, supporting our hypothesis that the field onset is linked to the effective gap of the TNLs.

H. Residual Resistance Ratio

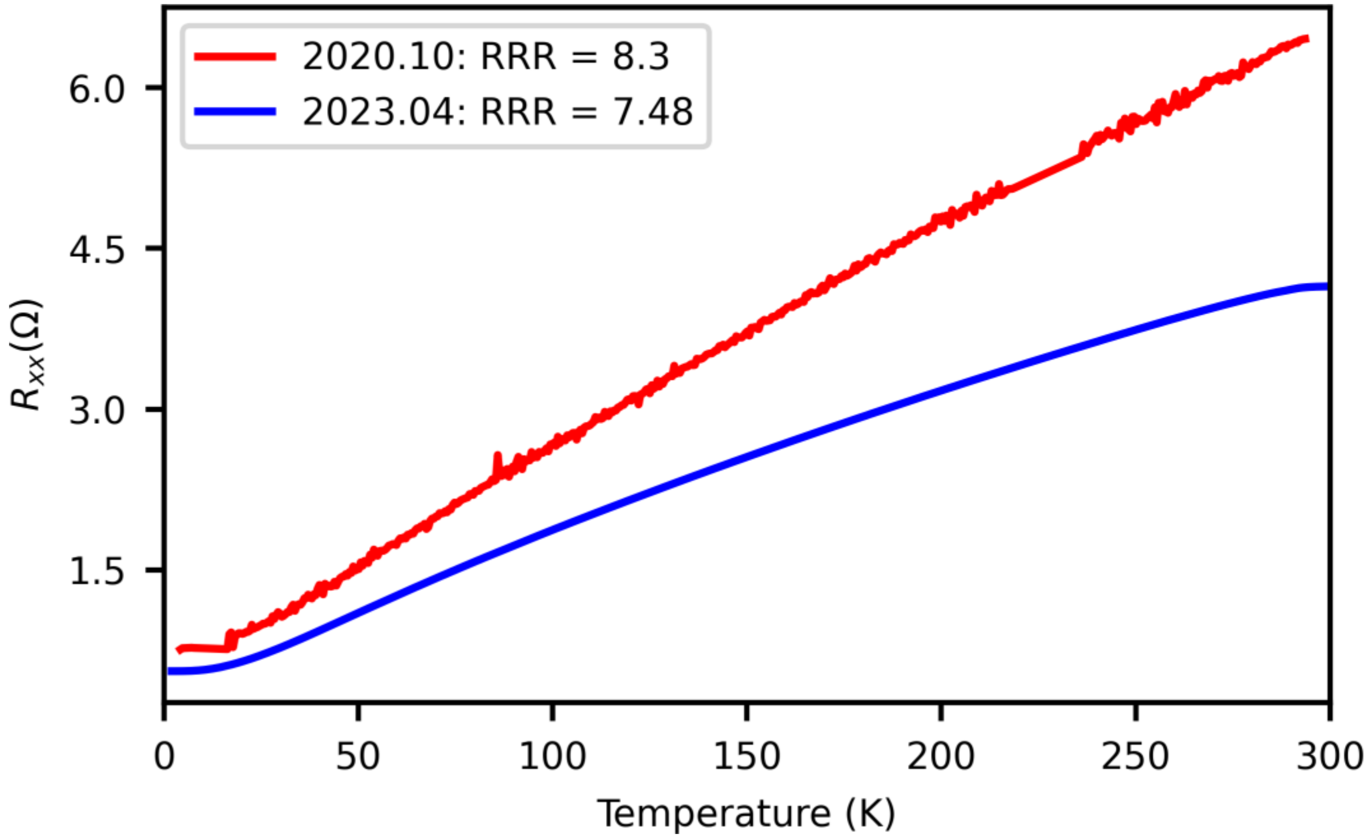

Supplementary Figure 9. Comparison of the longitudinal resistance of sample $D1$ between two cooldowns from room temperature to 5K. The residual resistance ration $RRR = \frac{R(300K)}{R(5K)}$ is indicated in the inset.

Fig. 9 shows a comparison of the longitudinal resistance of sample $D1$ between two cooldowns from room temperature to 5K, in 2020 (red, see Ref.[2]) and in 2023 (blue, this measurement run). The residual resistance ration $RRR = \frac{R(300K)}{R(5K)}$ is similar between the two cooldowns, and only decreased slightly in 2.5 years, showing the stability of the samples in air.

I. The Weyl point geometry of band 48

We focus on the band number 48, which when being the highest fully occupied band yields the correct number of electrons of the system. Of course the compound is not an insulator, however the Weyl points reported before are formed by band 48 and 49 and in the region $k_z \lesssim 0.17\frac{2\pi}{c}$ the highest occupied band is band 48.

As it turns out band 48 has more than the previously reported Weyl points. We group the Weyl points (WP) according to their averaged energy. We get six groups of six and three groups of twelve WPs with the exception of group G_8^{48}, see below. Within each group the WPs have individual $k_{x,y,z}$ coordinates of which the k_z positions are clustered very closely around $\pm|k_{z0}|$ with a group specific $|k_{z0}|$. The only (always fulfilled) true symmetry is ΘM_x. However, the deviations from the non-magnetic symmetries are small, such that the twelve-fold groups approximately follow $\{E,\Theta\}\,C_{3v}$, while the six-fold groups follow this symmetry if the WPs are assumed to lie exactly on the mirror planes. Group G_8^{48} is an exception in that it shows cross-over behavior between a 12-fold and a six-fold case as a function of increasing magnetic field. From the symmetry and from the small displacements shown in Fig. 10, Fig. 11 and Fig. 12 it is clear that the twelve-fold groups survive the transition to $B=0$, while the six-fold groups must dissolve in the nodal loops on the mirror planes.

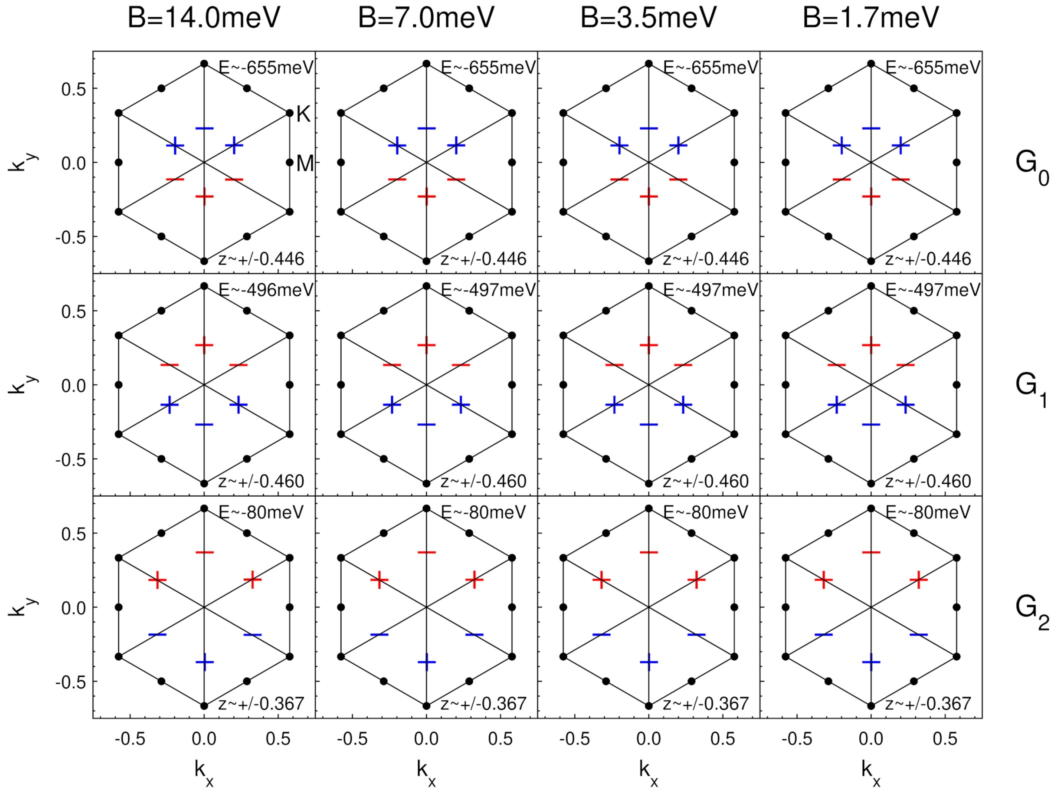

Supplementary Figure 10. WP groups 0, 1 and 2 of band 48. The average energy and k_z-position is denoted. Plus/minus signs denote the sign of the k_z-position and red/blue mark positive/negative chirality, resp.

The cross-over behavior of group 8 demonstrates both the survival of the twelve-fold groups as well as the dissolution of the six-fold groups. In Fig. 13 we follow the evolution of the three WPs close to the positive y-axis of Group G_8^{48} (Fig. 12 lower right panel). For high field ($B=14$meV) we have a six-fold group (in this case shifted to the positive k_x-side of the mirror). At around $B=3.5$meV a WP dipole emerges on the negative k_x-side of the mirror, whose separation increases with decreasing field (Fig. 13). Approaching $B=0$ the dipole part with equal chirality as the single WP from the six-fold subgroup is dissolved into the nodal loop, while the dipole part with opposite chirality to the single WP approaches the mirror symmetric position of the single six-fold WP, forming a fully symmetric twelve-fold group for $B=0$. Apparently the emergence of the WP dipoles happens at different fields for the six points of the high-field six-fold group (last row of Fig. 12) as allowed by the magnetic symmetry. We want to stress that there is no symmetry which would force the appearance of six-fold or twelve-fold groups as already indicated by the

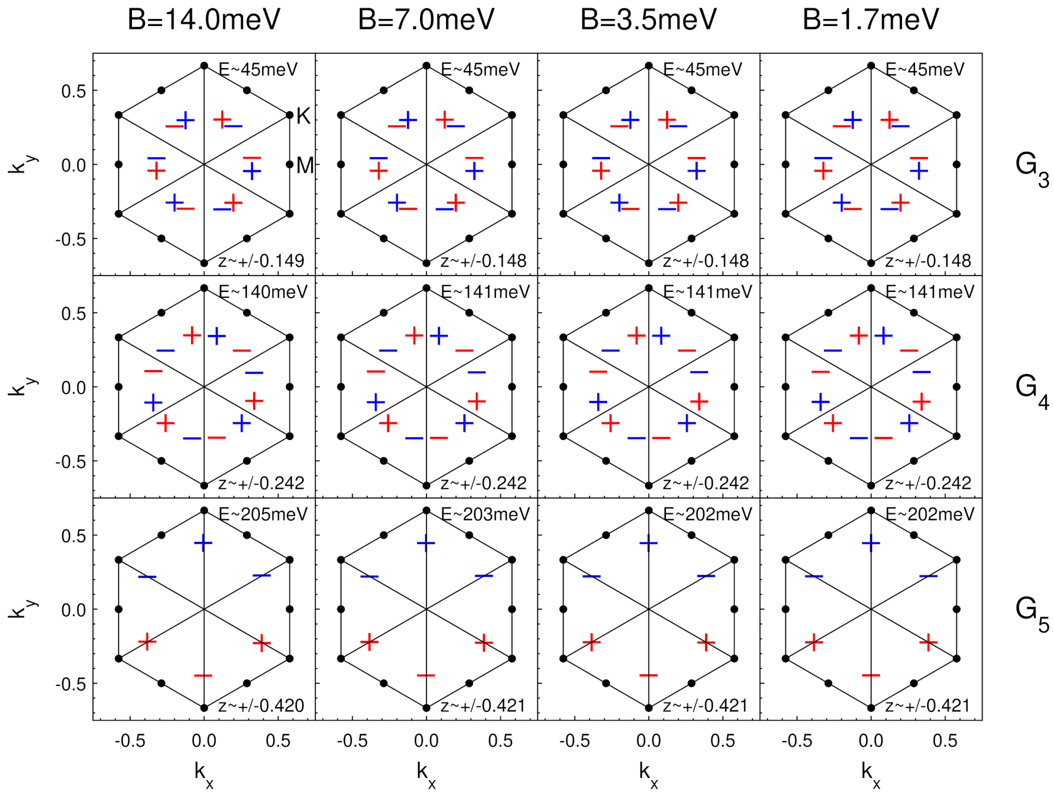

Supplementary Figure 11. WP groups 3, 4 and 5 of band 48. Group 3 is the one close to the Fermi level. The average energy and k_z-position is denoted. Plus/minus signs denote the sign of the k_z-position and red/blue mark positive/negative chirality, resp.

behaviour of G_8^{48}. A group could have any even number of points as long as they occur in pairs as imposed by the magnetic symmetry.

It turns out that all but groups $G_{3,4}^{48}$ consist of at least some type II Weyl points, while these two groups are formed only by type I points.

J. Chern signal

The Chern number of a k_z-plane is defined as

$$c\left(k_z\right)=\frac{1}{2\pi}\int dk_x dk_y F_z\left(\boldsymbol{k}\right) \tag{6}$$

with $\boldsymbol{A}\left(\boldsymbol{k}\right)=\sum_n\left\langle u_n\mid i\nabla_{\boldsymbol{k}}u_n\right\rangle$, $\boldsymbol{F}\left(\boldsymbol{k}\right)=\nabla_{\boldsymbol{k}}\times\boldsymbol{A}\left(\boldsymbol{k}\right)$, while the total anomalous Hall conductivity (AHC) is defined as

$$\begin{aligned}\Delta\sigma_{xy}&=-\frac{e^2}{\hbar}\frac{1}{\left(2\pi\right)^3}\int d^3kF_z\left(\boldsymbol{k}\right)\\&=-\frac{e^2}{\hbar}\frac{1}{\left(2\pi\right)^2}\int dk_z c\left(k_z\right)\\&=-\frac{e^2}{\hbar}\frac{1}{c2\pi}\int_0^1 dz c\left(z\right)\end{aligned}$$

with $c=6.162$Å and conversion constant

$$\frac{e^2}{\hbar}\frac{1}{c2\pi}=628.71\mathrm{S/cm} \tag{7}$$

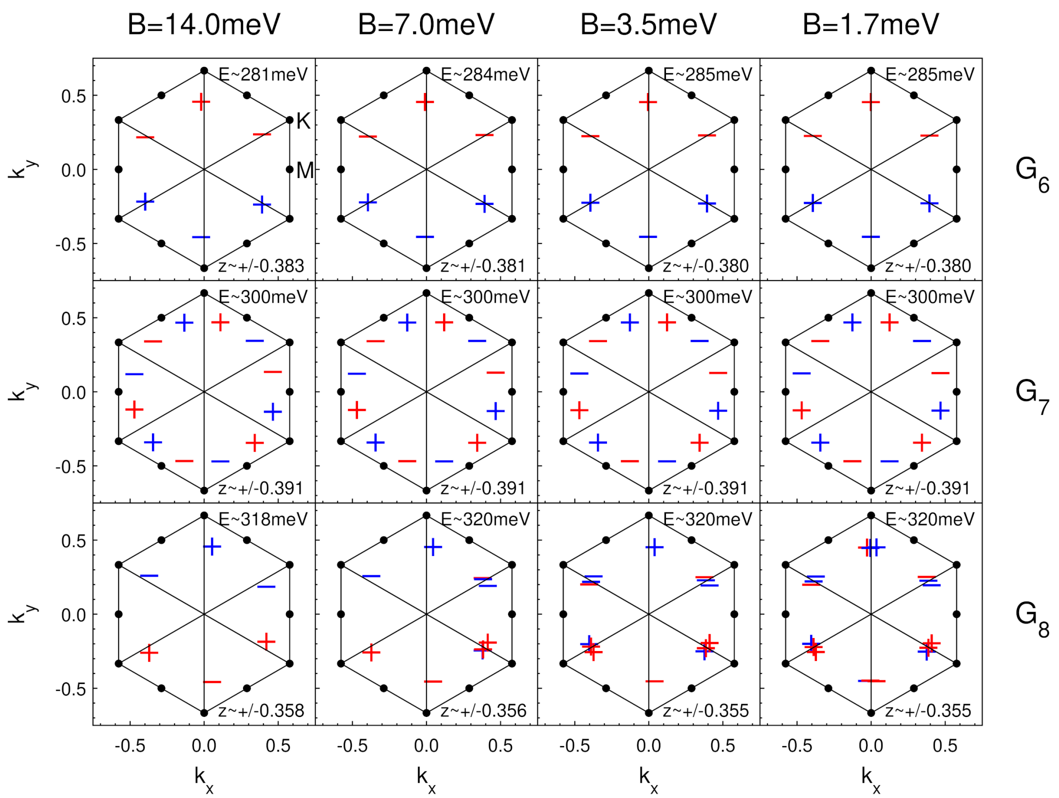

Supplementary Figure 12. WP groups 6, 7 and 8 of band 48. The average energy and k_z-position is denoted. Plus/minus signs denote the sign of the k_z-position and red/blue mark positive/negative chirality, resp.

The Chern signal $c\,(k_z)$ can be obtained by a gauge invariant plaquette type integral [3], which has better convergence properties than a simple sum-type integral, but is only valid for a constant number of occupied bands, i.e. an insulating k_x, k_y-plane. $c\,(k_z)$ is given by the sum of $\tilde{c} = \frac{1}{2\pi} i \ln U_1 U_2 U_3 U_4$ over all plaquettes of the planar Brillouin zone perpendicular to k_z with $U_i = \frac{\det\langle u_{k_i} | u_{k_{i+1}} \rangle}{|\det\langle u_{k_i} | u_{k_{i+1}} \rangle|}$, where k_i are the k-points at the four plaquette corners. The determinant is taken of the overlap matrix formed by the 48 (homo) lowest bands. The minimally needed mesh fineness is dictated by the WP separation in the k_x, k_y plane and the mesh fineness of the k_z-interval by the k_z-separation of the individual WPs, which turns out to be demanding. Additionally, the experimentally used magnetic fields ($\boldsymbol{B}$) are small from a band structure perspective and the subsequent WP shifts (and emergence of new WPs) leads to strong variations of $\boldsymbol{F}$ over rather small volumes.

To incorporate a Fermi energy eq. 7 is evaluated via a simple Riemann sum.

The Chern signal for the insulating case is entirely determined by the Weyl positions and hence the numerical results can be checked analytically. Each WP contributes a jump

$$c_{\mathrm{WP}}\,(k_z) = \chi_{\mathrm{WP}} \Theta\,(k_z - k_{z,\mathrm{WP}})$$

The integral over k_z gives the contribution $\Delta\sigma_{xy,\mathrm{WP}}\,(k_z) \propto -\chi_{\mathrm{WP}}\,(k_z - k_{z,\mathrm{WP}})\,\Theta\,(k_z - k_{z,\mathrm{WP}})$. The sum over all Weyl points for large k_z (total signal) then is

$$\Delta\sigma_{xy} \propto \sum_{\mathrm{WP}} \chi_{\mathrm{WP}} k_{z,\mathrm{WP}} \tag{8}$$

For a subgroup of six WPs with vanishing total chirality around some positive k_z (half of a twelve-fold group) $\Delta\sigma_{xy}$ becomes a function of the k_z-separation of the individual WPs. It turns out that the WPs are ordered in three pairs of two points with opposite χ. Hence, each pair contributes an up-down jump combination (Fig. 14.C) with $\Delta\sigma = \chi \Delta k_z$ which is proportional to the separation of the points in the pair (up to some global integer, which is determined by

the numerical integrals). The separations Δk_z are small and in leading order a linear function of the field B (Fig. 14.A). Additionally, for group G_3^{48}, the separations Δk_z together with their χ compensate the linear field dependence of Δk_z completely, which results in very tiny total $\Delta\sigma_{xy}$ values, which depend quadratic on B (Fig. 14.B).

The χ-uncompensated three WP subgroups at positive k_z of a six-fold group give an effective jump $\Delta c = \pm 1$ at the group's averaged k_z-position. Since, the k_z positions of the groups can have sizable distances these well separated jumps contribute large values to $\Delta\sigma_{xy}$, in fact much larger than the actual result including the Fermi surface (black line) in Fig. 3of the main text and Fig. 16 (keep in mind the conversion factor eq. S8).

The total result for $c\,(k_z)$ for four different fields B is shown in Fig. 15. Clearly the twelve-fold groups $G_{3,4,7}^{48}$ have compensating jumps within themselves and the separation of the individual jumps within such a group diminishes with diminishing field (Fig. 14.A). Each of these groups contributes a signal similar to the green line in Fig. 14.C. In contrast, the six-fold groups contribute effective jumps $\Delta c = \pm 1$ at different k_z positions each consisting of three closely spaced up-down-up or down-up-down jumps, whose internal separation diminishes with B, effectively becoming a single jump. Since the k_z positions of the group do not move around much with field, different groups do not compensate each other for $B \to 0$. The corresponding signal comes into existence immediately when $B > 0$, where these groups emerge out of the nodal loops. This situation is reminiscent of a quantum hall situation.

The discussion up to now was for a hypothetical insulating case where all bands up to the homo (48) are considered occupied. This means that all WPs of all groups are occupied exactly up to the touching point of the linear dispersion. This leads to the vertical jumps shown in Fig. 3of the main text, Fig. 14.C and Fig. 15. If a Fermi level μ is introduced close enough to the energy of a WP (or WP group for that matter), such that the linear dispersion still holds, the jumps get modified:

$$c_{\mathrm{WP}}\,(k_z) = \chi_{\mathrm{WP}}\Theta_\mu\,(k_z - k_{z,\mathrm{WP}})$$

with

$$\Theta_\mu\,(k_z) = \mathrm{sign}\,(k_z)\begin{cases}1 & |\mu| < |k_z| \\ \frac{|k_z|}{|\mu|} & |k_z| < |\mu|\end{cases}$$

and μ measured relative to the degenerate point and k_z being scaled with a suitable band velocity (omitted). Here we integrated over the whole infinite k_x, k_y-plane. Finite integration over a disk of radius R modifies the expression but not in an essential way. The modification is due to the necessary omission ($\mu < 0$) are addition ($\mu > 0$) of a disk of radius μ around the planar WP position and holds for $\mu \lessgtr 0$. The curve is essentially a step function with a linear rise of width $|\mu|$ instead of a vertical jump. The corresponding k_z-integral becomes

$$\Delta\sigma_{xy}\,(k_z) \propto \chi_{\mathrm{WP}}\Delta\sigma_\mu\,(k_z - k_{z,\mathrm{WP}})$$

$$\Delta\sigma_\mu\,(k_z) = \begin{cases}0 & k_z < -|\mu| \\ \frac{(k_z+|\mu|)^2}{4|\mu|} & |k_z| < |\mu| \\ k_z & |\mu| < k_z\end{cases}$$

which for k_z above $k_{z,\mathrm{WP}} + \mu$ gives the same total contribution of a WP group as eq. S9. The result is altered locally around the groups k_z-position but far away the AHC signal is Fermi level independent (see also [4]). This is of course only true if the ideal linear Weyl dispersion holds and if the broadening due to the linear sections does not become comparable to the size of the total k_z-interval. Interestingly, numerical results for Fermi levels close to a twelve-fold groups energy show a $c\,(k_z)$ signal of the group, which looks very similar to the idealized results derived here. For larger energy distances we see more and more deviations from the ideal behavior.

Fig. 14.D is the equivalent of Fig. 14.C for $\mu = E_{\mathrm{F}} = 45.3$meV. Note, how the vertical jumps become linearly interpolated jumps. The linear regions of the two contributions around the middle of the k_z-axis are quite narrow, since these WPs have an energy close to μ, while the other four WPs have larger deviations of their energy from μ and hence wider linear regions. The resulting $\Delta\sigma_{xy}(k_z)$ signal looks quite different from the constant homo case but its total value for large k_z is Fermi level independent.

In a real band structure the ideal WP linear dispersion bends into the real bands away from the WP position, which partially removes the Fermi level independence and leads to the washing out of the effects of the integer Chern numbers. Additionally, some of the WPs are highly anisotropic, which leads to further deviations from the idealized model discussed above.

This Fermi level independence gets also altered if the Weyl points are of type II, in which case the formulas above need to be modified and additionally the associated Fermi pockets, especially if they are small, quickly lead to a deviation from an ideal Weyl Hamiltonian.

K. Band 47

Unsurprisingly, the magnetic field also induces Weyl points in other bands. For k_z-values between 0.17 and 0.5 there are several Fermi surfaces sheets and hence the focus on the integer contribution of band 48 (which is the homo for the originally discussed Weyl point group (G_3^{48})) is not justified, especially for the peaks P_1 at $k_z \approx 0.27$, P_2 at $k_z \approx 0.38$ and P_3 at $k_z \approx 0.43$, which give the largest contribution to the AHC signal (black curve in Fig. 3and Fig. 16.A,B).

We hence have to analyze band 47 as well. It turns out that there are 11 Weyl point groups, which all are at least partially type II. All groups seem to be associated with nodal lines (See Fig. 16.C). Some contain only two or four Weyl points while the rest are six-fold, which means that all but the four-fold groups are uncompensated contributing an effective jump of the Chern number by ± 1. The groups with less than six points are consistent with the numerical plaquette type results (green curve in Fig. 16.B), which does however not exclude the possibility that we missed some Weyl points, which form compensating pairs and hence do not show in the numerical results at least in the used resolution.

Groups $G_{5\ldots 10}^{47}$ have energies between -100 and +125 meV. Fig. 16.AB show the comparison between the integer Chern signals for an assumed constant homo 48 and 47 as well as $c(k_z, E_F = 45.3\text{meV})$ and the resulting AHC signal. Groups $G_{1\ldots 4}^{47}$ have $k_z < 0.15$ and hence lay in the region where band 48 is the homo. Consequently, their Weyl points are fully occupied and the integer Chern signal is not realized if a Fermi energy is included.

Since, the groups mostly contain type II Weyl points the simple argument of energy independence of the AHC signal (for an ideal Weyl Hamiltonian) does probably hold to a lesser extend. The inevitable associated Fermi pockets lead to a quick deviation from the ideal case. It is therefore better to explain the large peaks $P_{1,2,3}$ of $c\,(k_z, E_\text{F} = 45.3\text{meV})$ via close by Weyl points. We managed to attribute peak P_2 at $k_z \approx 0.38$ (and probably also peak P_3 at $k_z \approx 0.43$) to the sixfold group G_8^{47} of band 47, which has Weyl point energies $+66\ldots+91$ meV. Fig. 17.A-C show the contribution $c\,(k_{x,y}, k_z)$ to the Chern signal $c\,(k_z)$ for the chosen Fermi energy of 45.3meV for three k_z-values in the upper panels and the highest occupied bands as function of $k_{x,y}$ for this Fermi level in the lower panel. The type II Weyl points of G_8^{47} are marked by red (positive chirality) and blue (negative chirality) pluses.

Fig. 17.D shows corresponding data at the approximate Weyl point position $k_z = 0.338$ and energy 78.5meV of G_8^{47}. The Weyl points sit where the pockets of band 48 touch the border between bands 46 and 47, which is a signature of a type II Weyl point. At these parameters no big Berry curvature can be seen. If we lower the energy to 45.3meV and shift k_z to 0.36 (Fig. 17.A) the pockets of band 48 get smaller and have nearly vanished for $k_Z = 0.373$ (close to the maximum of peak P_2), while at the same time Berry curvature gets induced at this very spot. For illustration the positions of the Weyl points of G_8^{47} are depicted in Fig. 17.E in the first BZ. They all lay close to a mirror plane (solid lines) and nodal lines.

Apparently, Weyl points need not be close in k_z-E-position to where the Berry curvature acquires a sizable signal. For peak P_1 we can establish a (linear) path through k_z-E-space from the position of the maximum of P_1 (Fig. 18.A) to the approximate Weyl point position (Fig. 18.G) of G_{10}^{47} ($k_z \approx 0.325$, $E = 130$meV), along which the topology of the Fermi surface sheets changes little, which makes the attribution of the Berry curvature of peak P_1 to group G_{10}^{47} likely. Since the WPs of G_{10}^{47} are not sitting at the exact same k_z and energy the Lifshitz transitions of the associated pockets (type II Weyl points) are not happening simultaneously along the path. Nevertheless, the transitions are clearly discernable. Note, that the WP chirality is opposite to the sign of the Bery curvature maxima. This is is a consequence of the jump of χ at the WP position as a function of k_z. Depending on which side of the WP one takes a cut the sign of the Berry curvature pattern switches. It should also be noted that the Berry curvature towards the end of the path (Fig. 18.I), has the same Berry curvature sign pattern at the WPs as the WP's chirality, however with sizable Berry curvature in the triangular area close to $k_{x,y} = 0$. The symmetry of the curvature in this area seems to approximately follow the full $\mathcal{C}_{3v}$ symmetry (as opposed to the curvature at the WPs) and hence largely integrates to a small total signal.

It turns out that G_5^{47} has Weyl point position at very similar $k_{x,y}$ as G_{10}^{47} but at $k_z \approx 0.168$ and $E \approx -98$meV. However, in this case the Fermi surface sheets evolve into a completely different topology (Fig. 19). Again the Berry curvature pattern changes sign when going through the WPs, only this time the chirality pattern coincides with the BC pattern at peak P_1, which is due to the fact that G_5^{47} has an effective positive jump of the integer Chern number. Indeed the pockets between bands 46 and 47 are the positions which yield the BC maxima.

L. Lattice structure

We used space group 157 (P31m) with lattice parameters $a = b = 6.57316$Å and $c = 6.16189$Å. The Wyckoff positions are: Pt at $(0.2619, 0, 0.004)$, Bi_1 at $(0, 0, -0.359)$, Bi_2 at $\left(\frac{2}{3}, \frac{1}{3} - 0.204\right)$ and Bi_3 at $(-0.3856, 0, 0.271)$.

M. TNL conversion into Weyl Nodes: Model Hamiltonian

Our starting point is a $\mathbf{k}\cdot\mathbf{p}$ model for two nodal loops centered around two valleys $\Lambda_{1,2}$ related by time-reversal symmetry and located on a mirror invariant plane, which, as in the main part of the manuscript, we take as $k_x \equiv 0$. The corresponding vertical mirror symmetry can be represented as $\mathcal{M}_x = -i\sigma_x \otimes \tau_0$ with the Pauli matrix vectors $\boldsymbol{\sigma}$ and $\boldsymbol{\tau}$ that act in spin and valley space respectively. Since time-reversal symmetry exchanges the two valleys, it can be represented as $\Theta = -i\sigma_y \otimes \tau_x$. We next introduce a two band model Hamiltonian, which generalizes the model considered in Ref.[5], that respects time-reversal and mirror symmetry and reads:

$$\mathcal{H}_{\mathbf{k}\cdot\mathbf{p}} = \alpha k_x k_y \sigma_z \otimes \tau_z + \alpha k_x k_z \sigma_y \otimes \tau_z + \beta \left(k_0^2 + k_x^2 - k_y^2 - k_z^2\right) \sigma_x \otimes \tau_z. \tag{9}$$

For $k_x \equiv 0$ two circular nodal loops of radius k_0 are obtained. We next consider the effect of a planar magnetic field introducing a Zeeman coupling

$$\mathcal{H}_{Zeeman} = B\cos\theta\ \sigma_x \otimes \tau_0 + B\sin\theta\ \sigma_y \otimes \tau_0 \tag{10}$$

where θ indicates the angle of the planar magnetic field from the $\hat{x}$ direction. It is instructive to first consider the effect of a magnetic field with $\theta \equiv 0$, in which case the vertical mirror symmetry is preserved. For small enough (positive) values of the magnetic field, one finds that the nodal line in one valley expands – its radius is renormalized by the magnetic field to $\sqrt{k_0^2 + B}$ – while the nodal line in the opposite valley get shrunk to a radius $\sqrt{k_0^2 - B}$. At the critical value of magnetic field $B_c \equiv k_0^2$ the nodal line shrinks to a single point (see Figure 20.a and Figure 21.b). It is at this critical magnetic field that the conversion of the nodal line into Weyl points occurs. For $B > B_c$ zero energy states are in fact obtained at $k_y \equiv k_z \equiv 0$ and at the two mirror symmetry related momenta $k_x \equiv \pm\sqrt{B - k_0^2}$ (see Figure 20.a). The conversion by a mirror symmetry preserving planar magnetic field is therefore "local" in momentum space: a nodal line shrinks to a single point out of which two Weyl nodes of opposite chirality emerge.
The conversion of a nodal line into Weyl nodes is instead completely different in nature when considering a planar magnetic field that breaks the protecting vertical mirror symmetry. It in fact occurs even for infinitesimal B values. Let us take the planar magnetic field to be parallel to the vertical mirror plane and therefore oriented along the $\hat{y}$ direction. We thus set $\theta = \pi/2$ in the Zeemann Hamiltonian above. Using that the Hamiltonian is diagonal in valley space, the eigenenergies can be then written as

$$E^2 = \alpha^2 k_x^2 k_y^2 + \left(\alpha k_x k_z \pm B\right)^2 + \beta^2 \left(k_0^2 + k_x^2 - k_y^2 - k_z^2\right)^2 \tag{11}$$

where $\pm$ distinguishes the two valleys. The spectrum is symmetric under $E \to -E$. Therefore, Weyl nodes, if present, will appear as zero-energy states. These are determined by requiring that the three squared polynomials in the equation above must simultaneously vanish. From the first term on the r.h.s. of the equation above we immediately have that the Weyl nodes will sit on the $k_y \equiv 0$ plane. The two constraints $\alpha k_x k_z \pm B \equiv 0$ and $k_0^2 + k_x^2 - k_z^2 \equiv 0$ determine the complete position of two Weyl nodes per valley. We note that since the combined $\mathcal{M}'_x$ point group symmetry is preserved the Weyl nodes in opposite valleys are paired up to have opposite k_z values and equal k_x values. These results are sketched in Figure 20.c. For $B \to 0$ we find that the Weyl nodes appear at $k_x \simeq 0$ and $k_z \simeq \pm k_0$. In other words, Weyl nodes emerge out of a nodal loop with a k_z distance that equals the nodal loop radius. This highlights the "non-local" conversion of nodal loops into Weyl nodes.

N. TNL gap in magnetic field and thermal activation

It is possible to estimate the size of the gap at the onset field $B_{ons} = 2.8$ T by considering that is equates the thermal energy: $-g\mu_B m B_{ons} = k_B T$ (with $m = \pm 1/2$ for electrons). For this, we must assume a value of the g-factor. With a g-factor of 6 for band 28 (from [6]), we get $-g\mu_B m B_{ons} \sim 486$ μeV, which is very consistent with $k_B T \sim 430$ μeV at $T = 5$ K.
We can compare this to the TNL gap from DFT calculations performed for Zeeman energies $E_Z = 500$ μeV and $E_Z = 1$meV, although some important precautions must me taken when comparing the two, as we will detail later. Figure 22 shows the points considered along the TNL in (a) and the gap along the TNL at both Zeeman energies in (b). The TNL is already gaped by about 500 μeV in some places at $E_Z = 500$ μeV, while at $E_Z = 1$meV almost the entire TNL is gaped by more than 500 μeV. This is consistent with the small calculation shown above.
This DFT calculation cannot however be directly compared to the real system for two reasons: First, the points shown are in the mirror plane $\mathcal{M}_x$), while the WNs do not lie in the plane (they move in the k_x direction under the magnetic field). Second, the TNL does not sit exactly at E_F, nor is it at a constant energy, meaning the temperature does not have the same effect in all parts of the TNL.

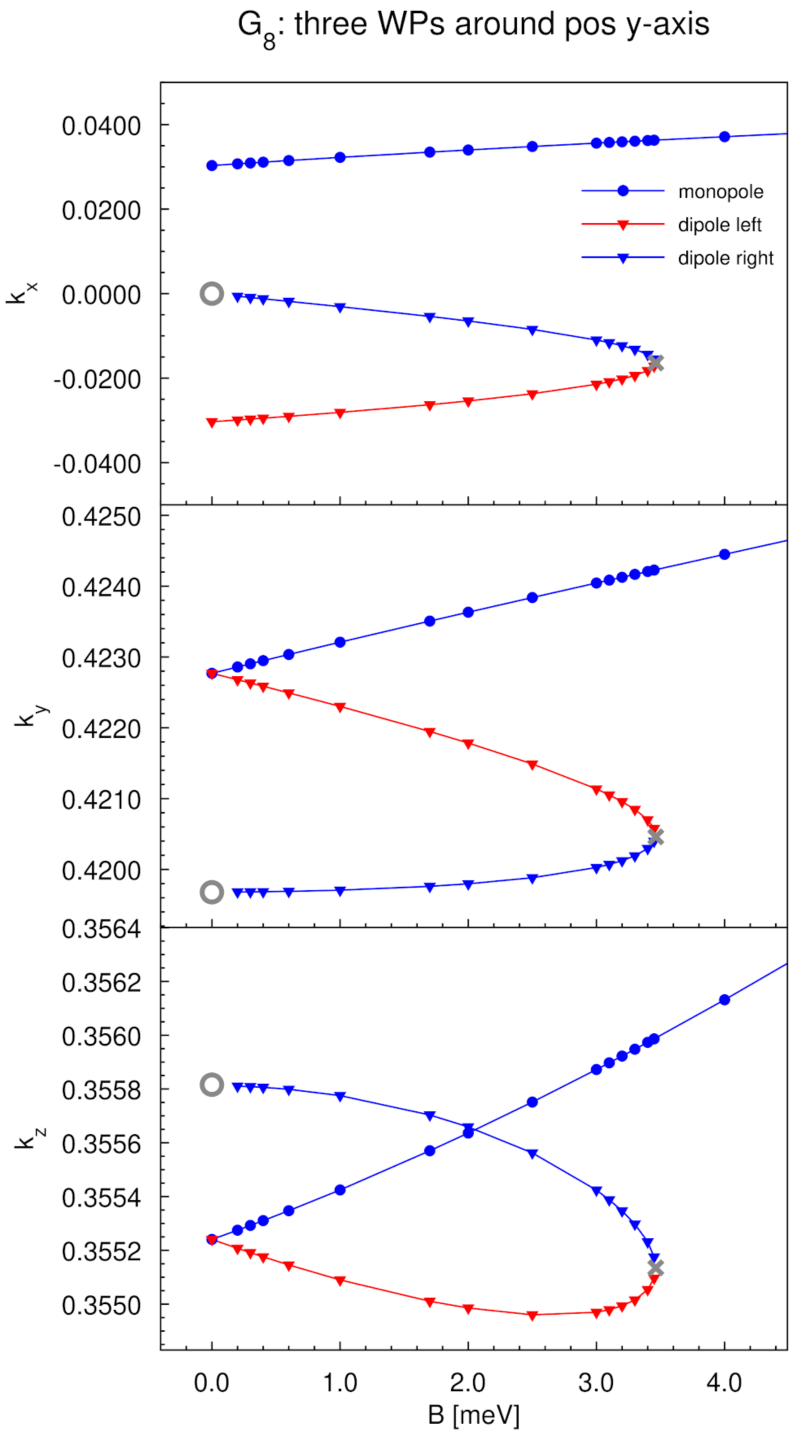

Supplementary Figure 13. Evolution of the three WPs close to the positive y-axis of G_8^{48} for small magnetic fields. The open circle denotes the position at which one of the WPs dissolves into the nodal loop. At $B = 0.35$ a dipole emerges at negative k_x (gray cross). Compare to last row of Fig. 12.

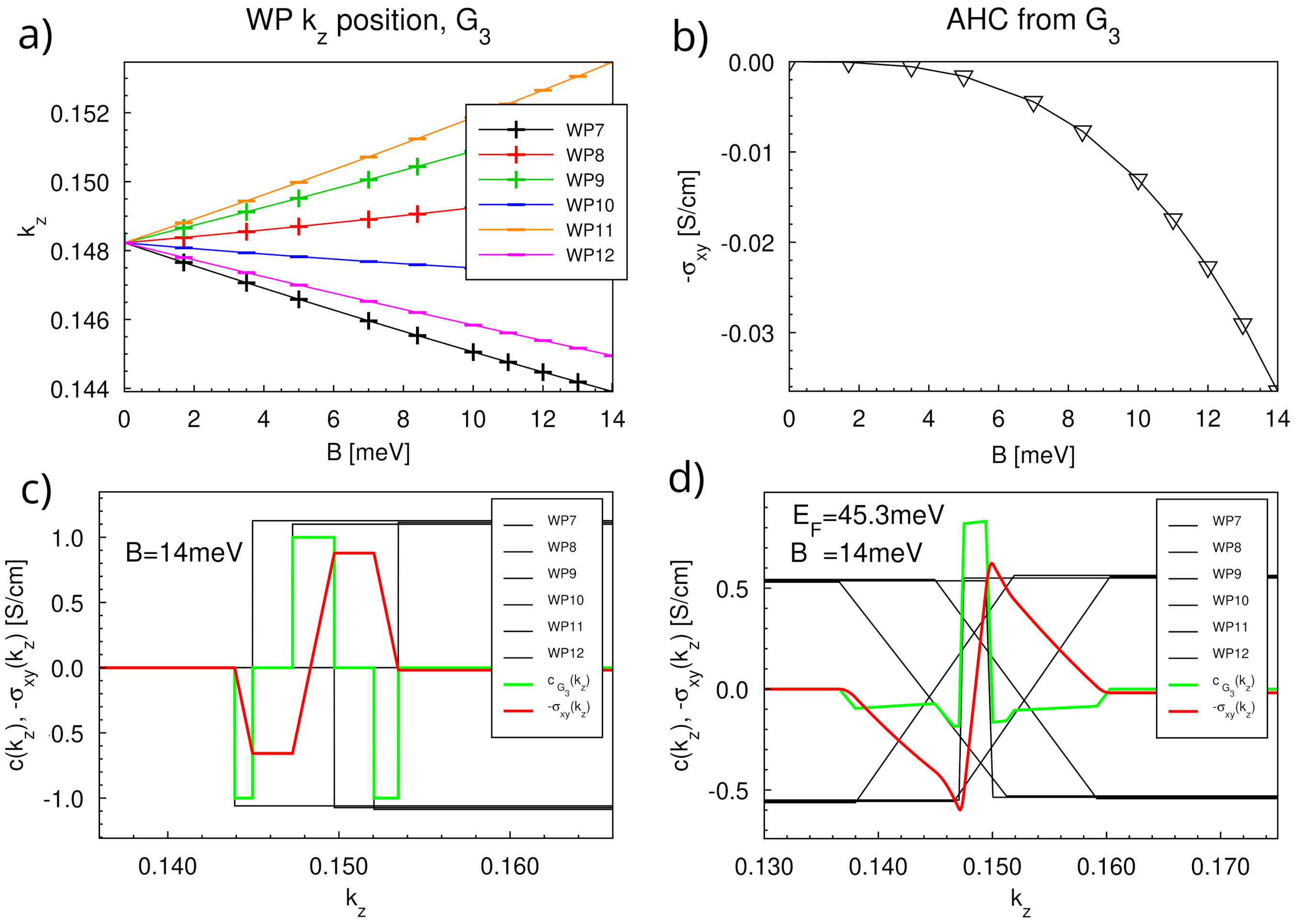

Supplementary Figure 14. a) the evolution of the WP k_z-position of the six WP with $k_z > 0$ of G_3^{48} as a function of B. The plus/minus symbols denote the chirality. b) The resulting AHC signal for the fully linearly compensated group G_3^{48} as a function of field B assuming ideal Weyl dispersion. c) The individual contributions (jumps) due to the six $k_z > 0$ WPs of G_3^{48} (black) offset along the y-axis for better visibility, their sum $c\,(k_z)$ and its k_z-integral $-\Delta\sigma_{xy}\,(k_z)$ in S/cm (red). d) As c) but for $E_\mathrm{F} = 45.3$meV.

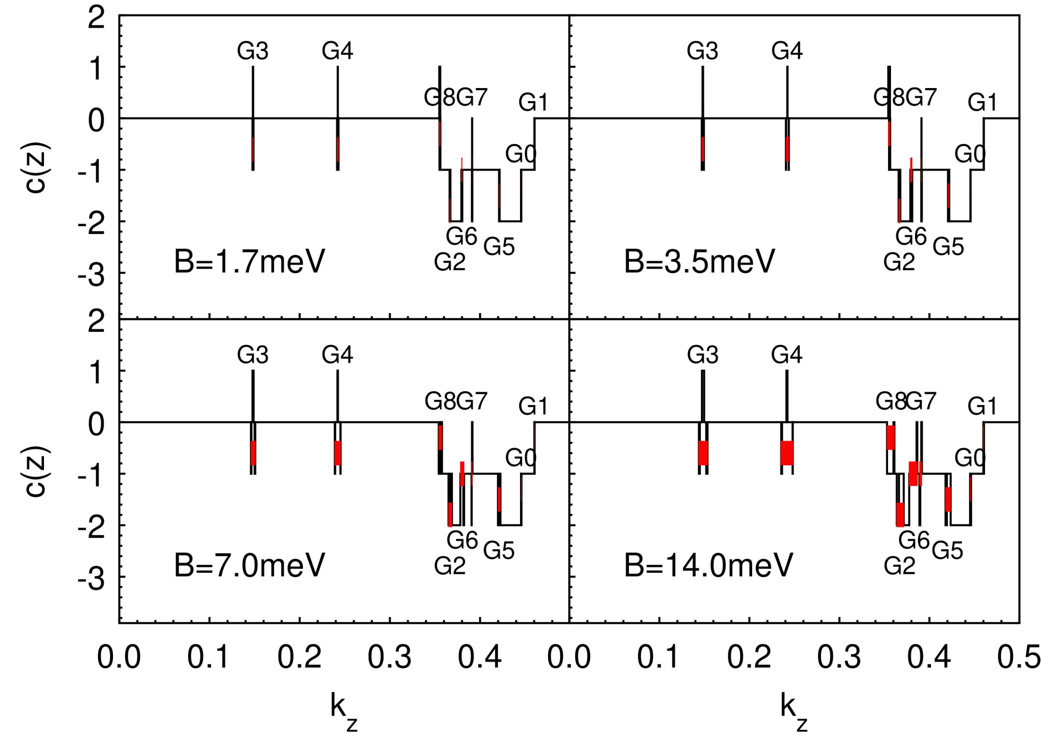

Supplementary Figure 15. Chern signal $c\,(k_z)$ for four magnetic fields for constant homo 48. The jumps are labeled by the corresponding WP groups. The red bars span all WPs of the corresponding group.

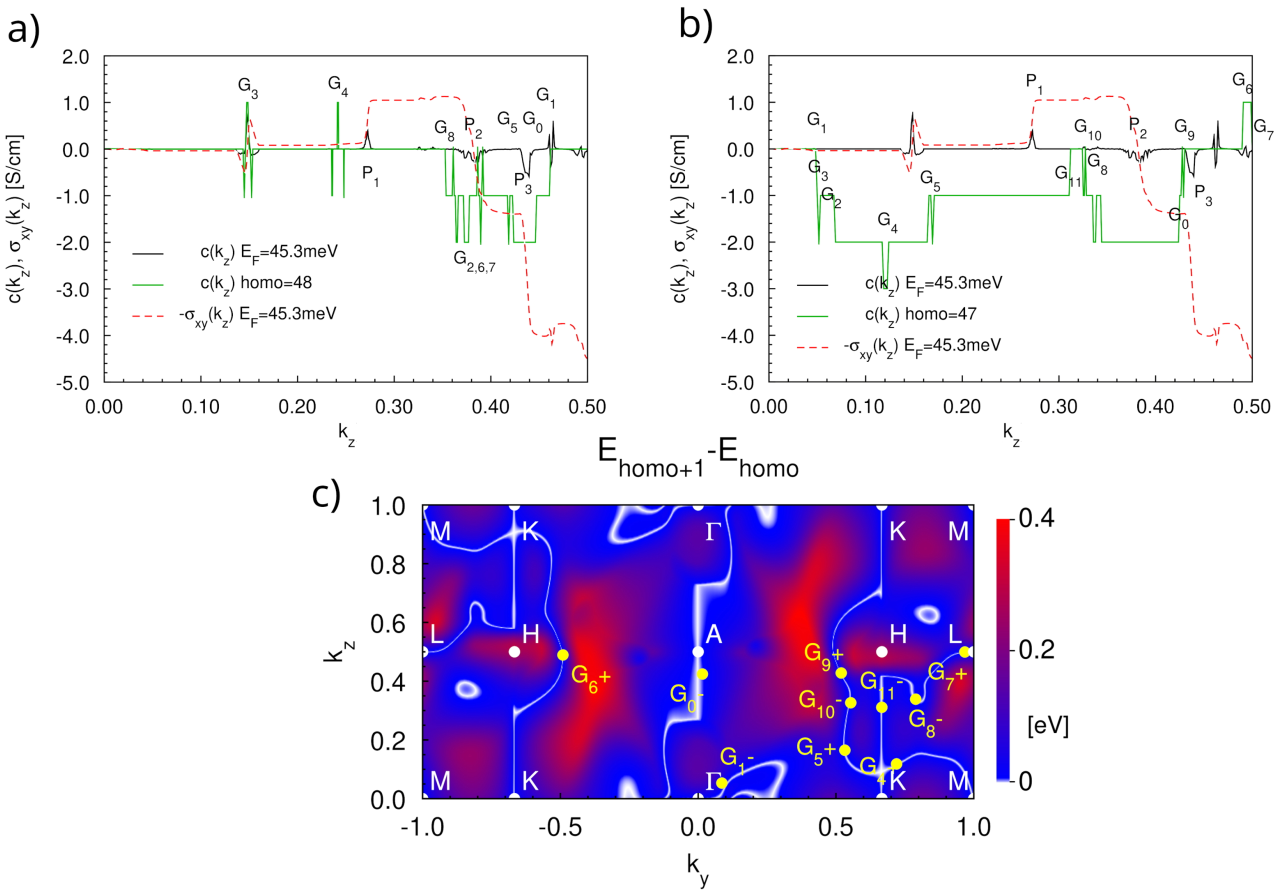

Supplementary Figure 16. **a,b**: the k_z-resolved Chern number for (green) constant homo, (black) constant Fermi energy and (red-dashed) the resulting AHC signal $-\Delta\sigma_{xy}(k_z)$ for **a**: homo 48 and **b**: homo 47. **c**: the Energy gap for homo 47. White denotes zero gap (nodal lines). All groups except $G^{47}_{2,3}$ are shown and fall close the nodel lines. $G^{47}_{2,3}$ are two-point groups and are closer to other mirror planes and hence do not fit into this picture.

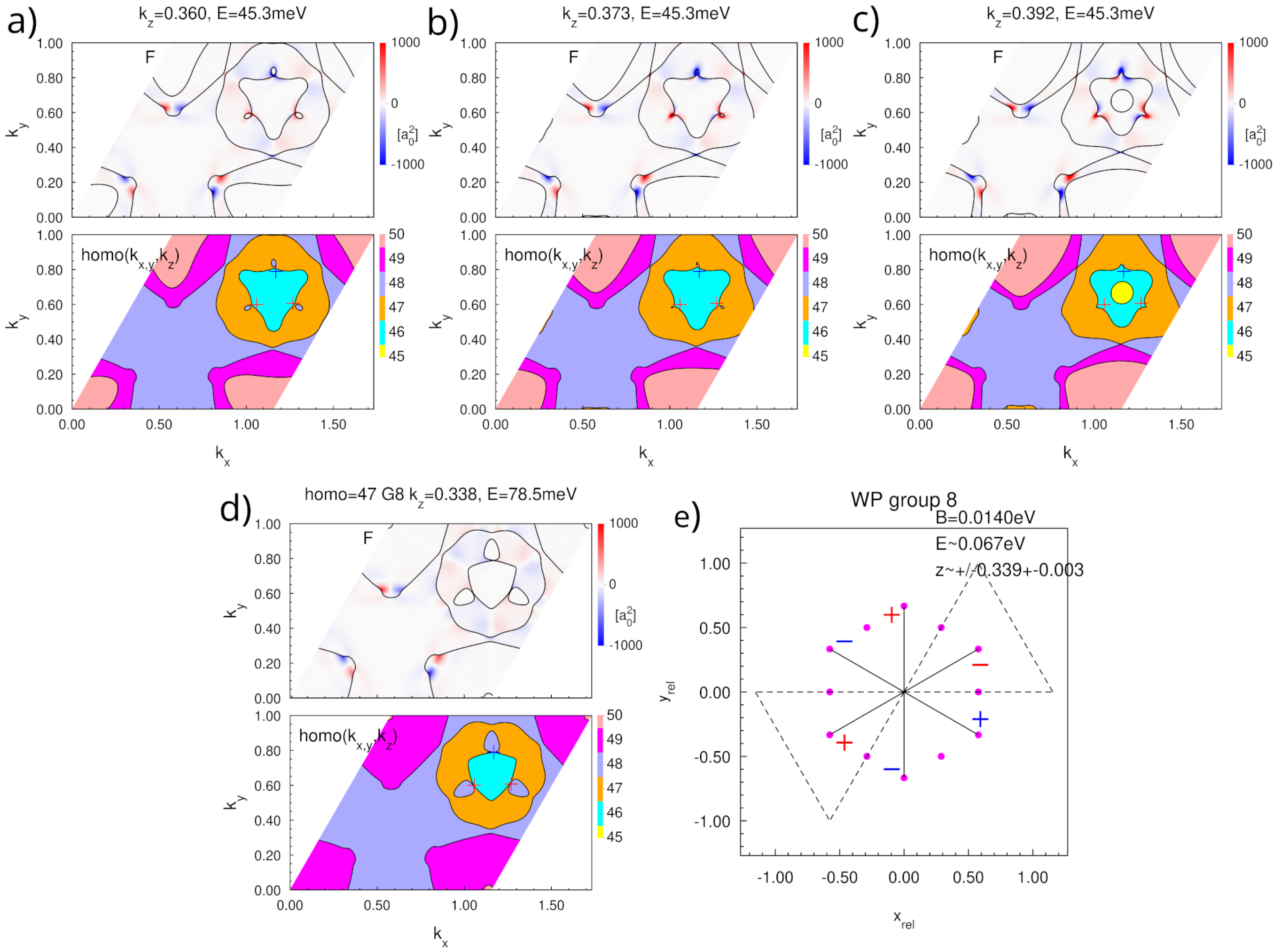

Supplementary Figure 17. **a-c**: $c\,(k_{x,y},k_z)$ (upper panel) and homo$\,(k_{x,y},k_z)$ (lower panel) for the peak P_2 at Fermi energy 45.3meV for three k_z values. **d**: Same data as in **a-c** at k_z and E of the sixfold group G_8^{47}. **e**: The arrangement of the Weyl points of G_8^{47}. The Weyl points are marked with pluses, their chirality with red(positive) or blue(negative) color.

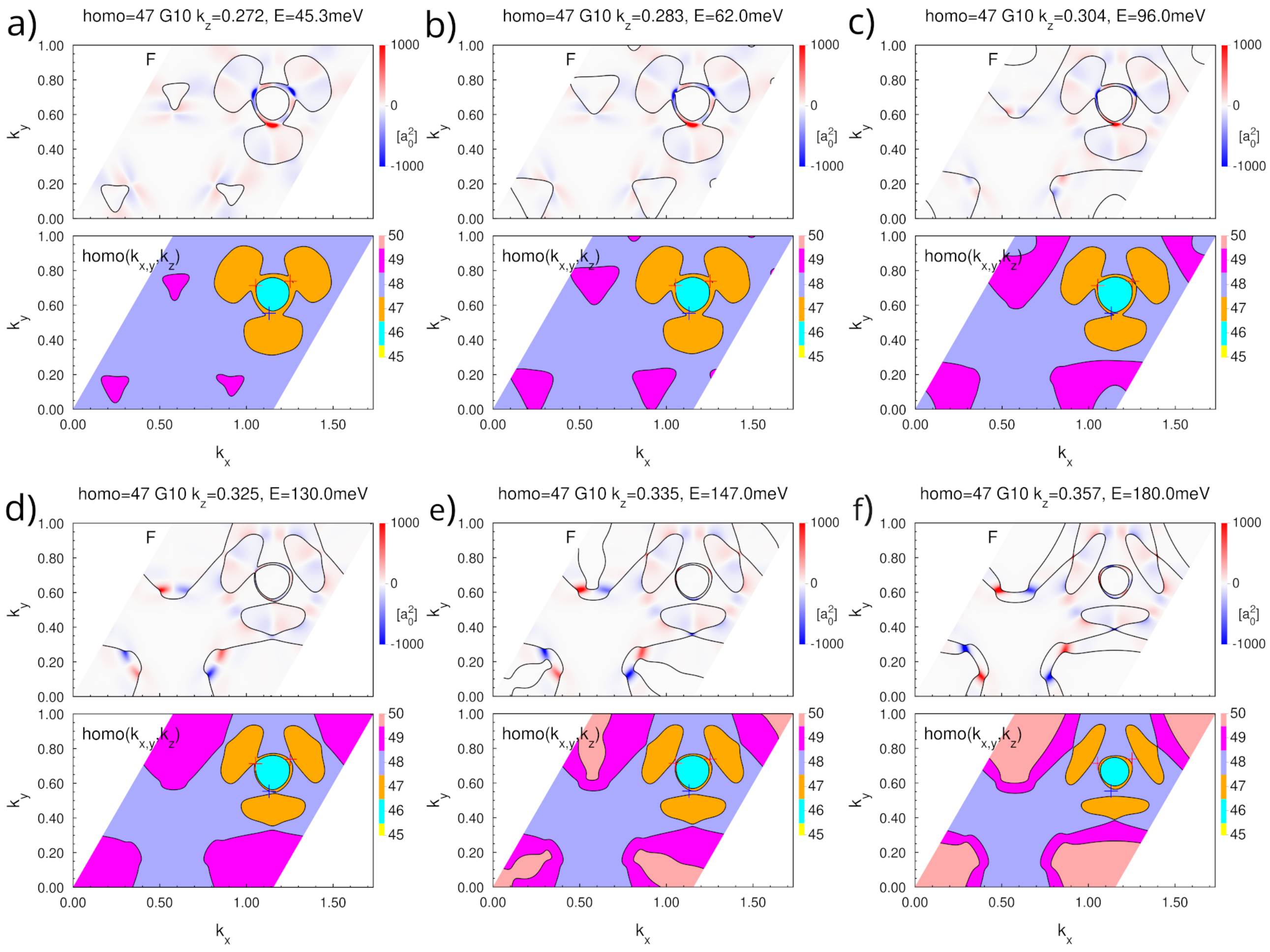

Supplementary Figure 18. Peak P_1. **a**: $c\,(k_{x,y}, k_z)$ and homo structure for $k_z = 0.272$ and $E_F = 45.3$meV (maximum of P_1). **b**…**f**: evolution of $c\,(\boldsymbol{k})$ along a linear path in k_z and E from P_1 (a) through the approximate WP k_z- and E-position of G_{10}^{47} (d) up to a position on the other side of the WP position (f) , with chirality indicated by red(positive) and blue(negative) color resp.

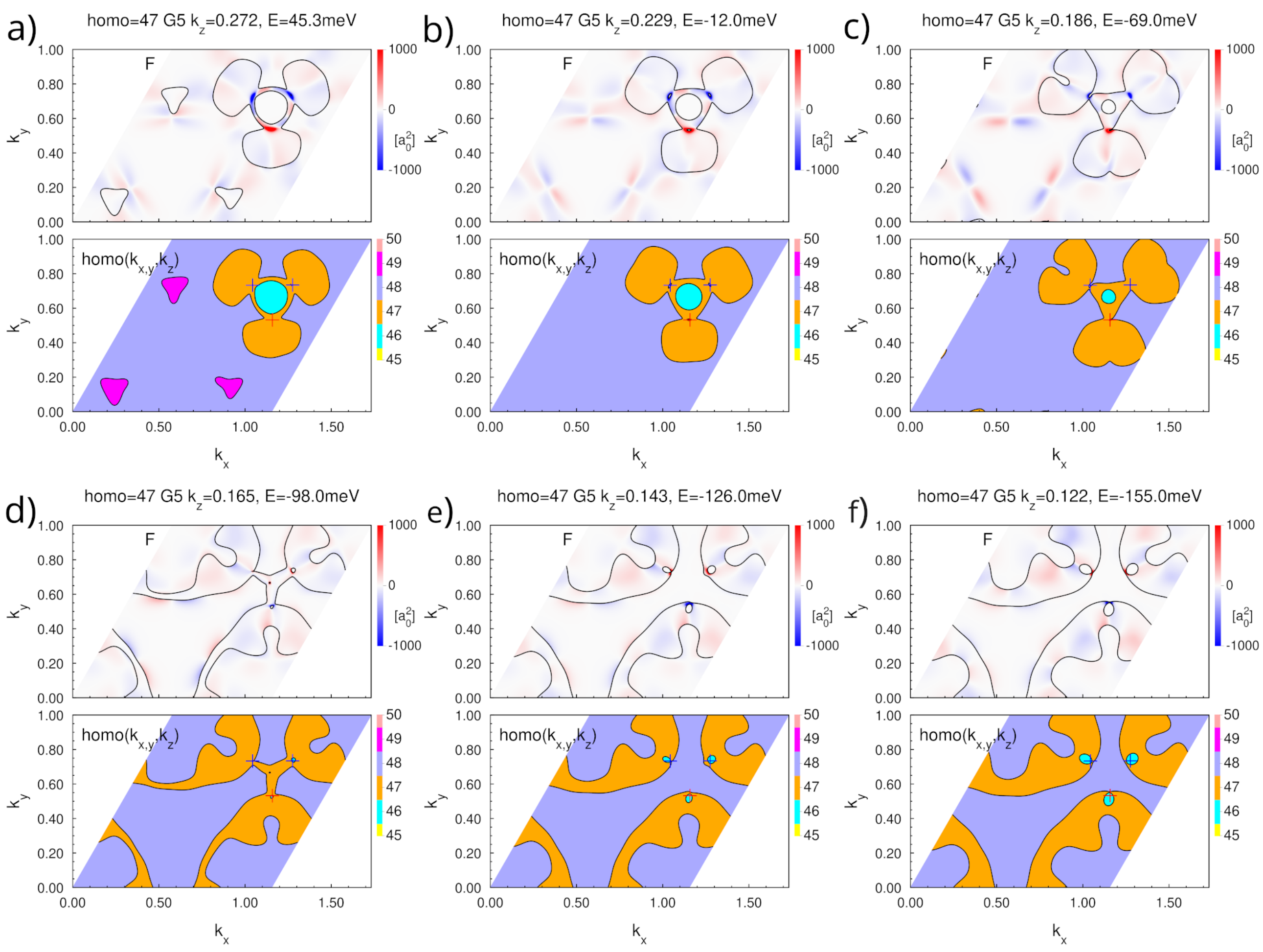

Supplementary Figure 19. Peak P_1. **a**: $c\,(k_{x,y}, k_z)$ and homo structure for $k_z = 0.272$ and $E_F = 45.3$meV (maximum of peak P_1). **b**. . .**f**: evolution of $c\,(\boldsymbol{k})$ along a linear path in k_z and E from peak P_1 (a) through the approximate WP k_z- and E-position of G_5^{47} (d) up to a position on the other side of the WP position (f). Pluses mark the position of the WPs, with chirality indicated by red(positive) and blue(negative) color, resp.

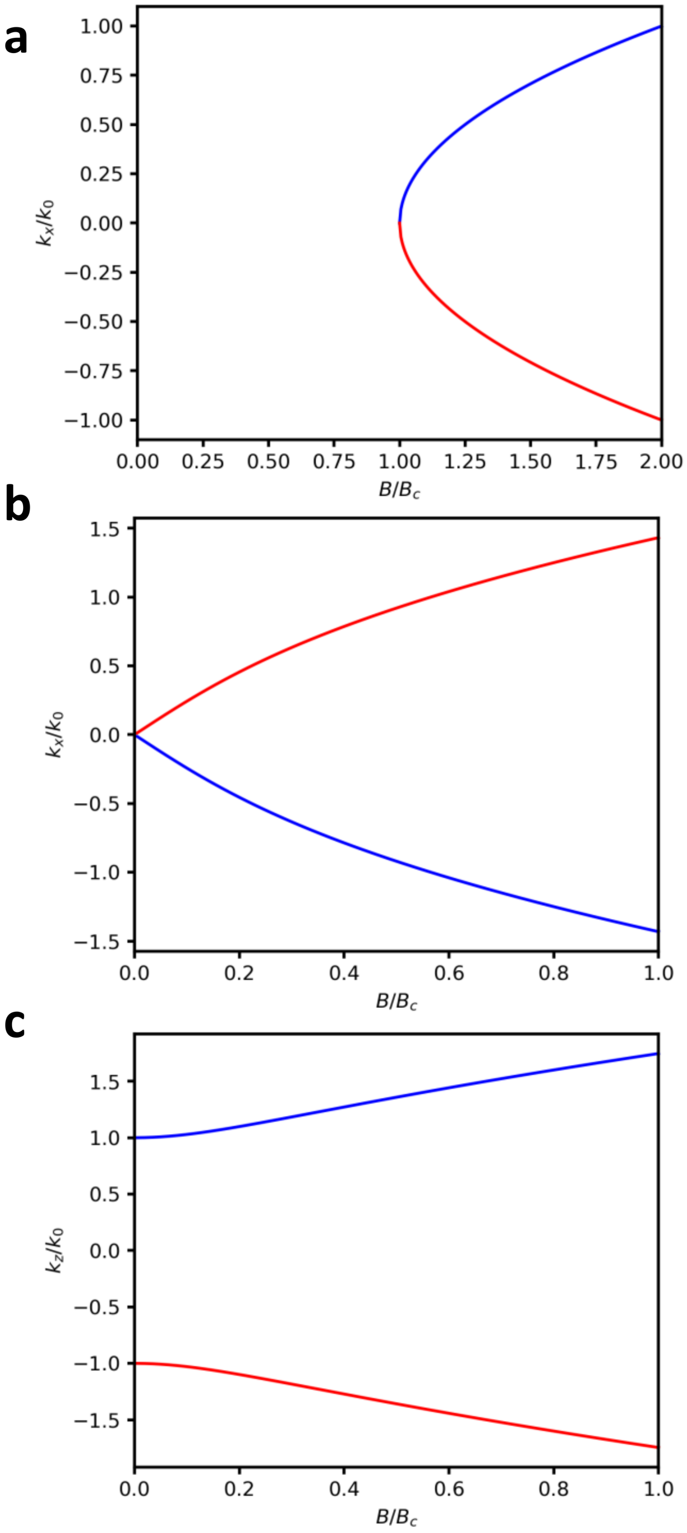

Supplementary Figure 20. Toy model showing the position of the Weyl nodes (originating from the TNL) as a function of a magnetic field which either preserves the $\mathcal{M}_x$ mirror symmetry **(a)**, or breaks that mirror symmetry **(b,c)**. In each graph, the positions of the two Weyl nodes of opposite chiralities are shown in blue and red, respectively. All WNs always sit in the $k_y = 0$ plane. **(a)** shows the k_x position of the two WNs when the field is along k_x. In this case, both WNs are always at $k_z = 0$. It can be seen that the two WNs originate from the same point at $k_x = 0$ when the field reaches the critical value. **(b,c)** show the k_x and k_z positions, respectively, of the two WNs when the field is along k_y. As soon as $B > 0$, two WNs appear at $k_x = 0$ and $k_z = \pm 1$, and start drifting in magnetic field. This shows the non-local nature of the WN creation from TNLs.

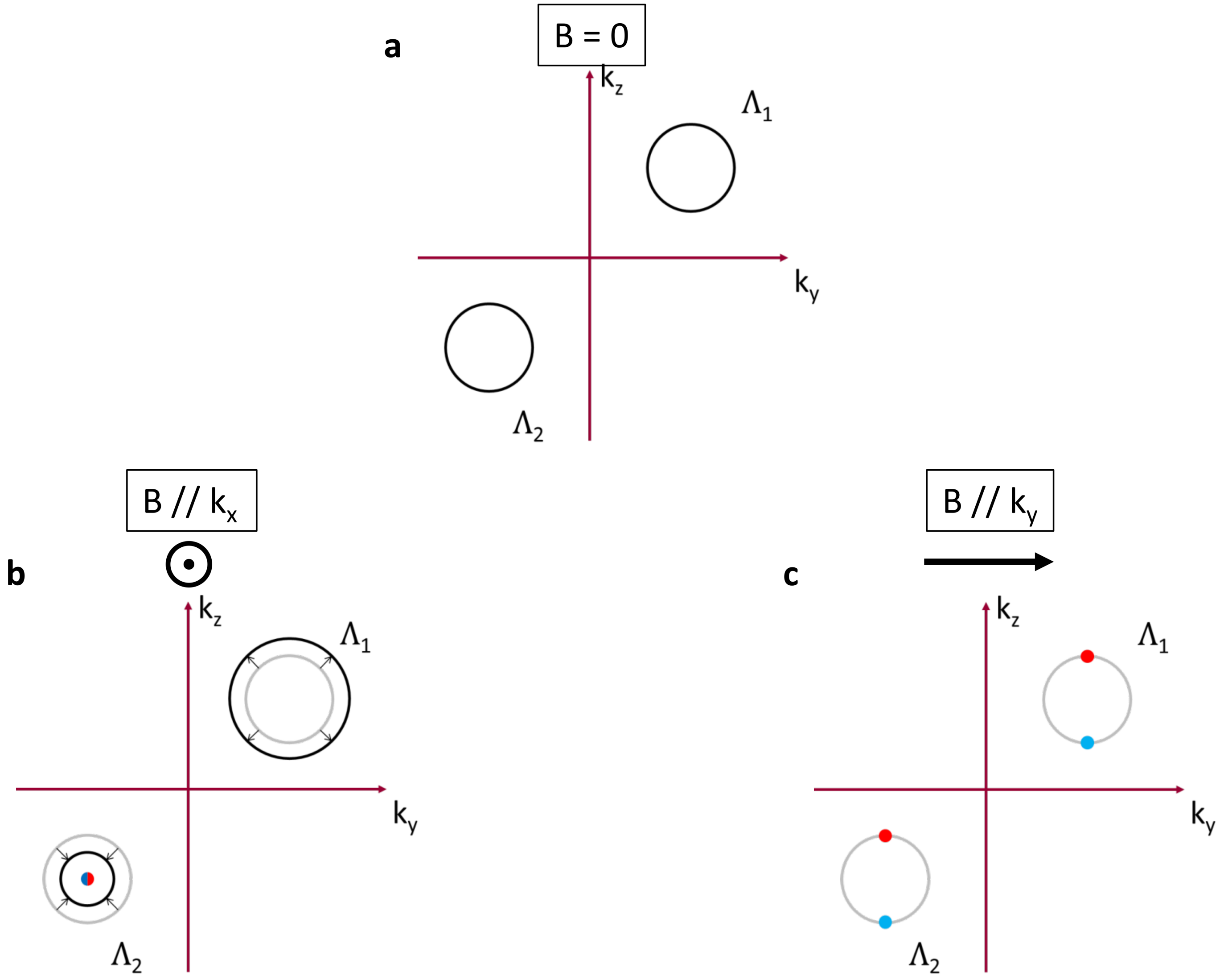

Supplementary Figure 21. Toy model showing the position of the Weyl nodes (originating from the TNL, shown in **(a)**) as a function of a magnetic field which either preserves the $\mathcal{M}_x$ mirror symmetry **(b)**, or breaks that mirror symmetry **(c)**. In each graph, the positions of the two Weyl nodes of opposite chiralities are shown in blue and red, respectively. All WNs always sit in the $k_x = 0$ plane. **a**: The two TNLs, in the $k_x = 0$ plane, in the absence of magnetic field. **b**: Evolution of the two TNLs when the field is along k_x, and preserves the mirror symmetry. One TNL expands, while the other one shrinks under the magnetic field. Above a critical magnetic field B_c, the shrinking TNL converts into two WNs of opposite chiralities at the same point. **c**: Evolution of the two TNLs when the field is along k_y, and breaks the mirror symmetry. Even at infinitesimal fields, the TNLs split into two WNs each. In this configuration, the pairs of WNs of opposite chiralities appear at maximal distance from one another.

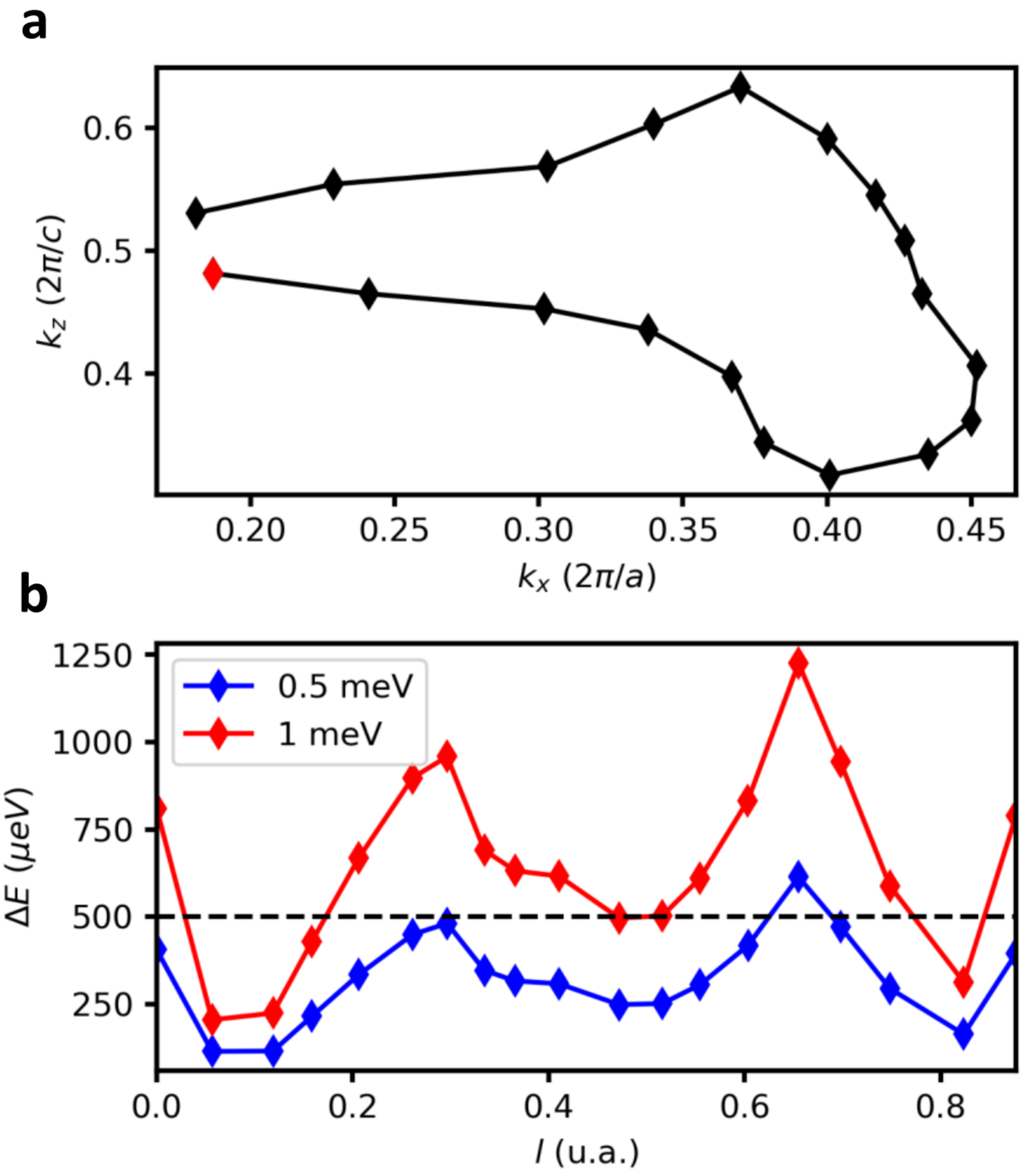

Supplementary Figure 22. DFT calculation of the gap opening in field at different points along the TNL. (a) Black diamonds: (k_x,k_z) positions of the different points considered along the TNL for the analysis (in the mirror plane $\mathcal{M}_x$); Red diamond: First point considered when going along the TNL. (b) Size of the gap along the TNL, for two Zeeman energies (0.5 meV in blue, and 1 meV in red). The parameter l corresponds to the length along the path going through all points in (a), starting from the red diamond.

[1] R. Battilomo, N. Scopigno, and C. Ortix, Physical Review Research **3**, 1 (2021).
[2] A. Veyrat, V. Labracherie, D. L. Bashlakov, F. Caglieris, J. I. Facio, G. Shipunov, T. Charvin, R. Acharya, Y. Naidyuk, R. Giraud, J. Van Den Brink, B. Büchner, C. Hess, S. Aswartham, and J. Dufouleur, Nano Lett. **23**, 1229 (2023).
[3] T. Fukui, Y. Hatsugai, and H. Suzuki, J. Phys. Soc. Jpn. **74**, 1674 (2005).
[4] A. A. Burkov, Phys. Rev. Lett. **113**, 187202 (2014).
[5] X.-Q. Sun, S.-C. Zhang, and T. Bzdušek, Phys. Rev. Lett. **121**, 106402 (2018).
[6] L. Xing, R. Chapai, R. Nepal, and R. Jin, npj Quantum Materials **5**, 10.1038/s41535-020-0213-9 (2020).
